# Long non-coding RNA NEAT1 mediated RPRD1B stability facilitates fatty acid metabolism and lymph node metastasis via c-Jun/c-Fos/SREBP1 axis in gastric cancer

**DOI:** 10.1186/s13046-022-02449-4

**Published:** 2022-09-29

**Authors:** Yongxu Jia, Qian Yan, Yinli Zheng, Lei Li, Baifeng Zhang, Zhiwei Chang, Zehua Wang, Hong Tang, Yanru Qin, Xin-Yuan Guan

**Affiliations:** 1 Department of Clinical Oncology, the First Affiliated Hospital, Zhengzhou University, Zhengzhou, China; 2 Department of Clinical Oncology, The University of Hong Kong, Room L10-56, Laboratory Block, 21 Sassoon Road, Hong Kong, China; 3 Department of Colorectal Surgery, Guangdong Institute of Gastroenterology, Guangdong Province Key Laboratory of Colorectal and Pelvic Floor Disease, The Sixth Affiliated Hospital, Sun Yat-Sen University, Guangzhou, China; 4 State Key Laboratory of Oncology in Southern China, Sun Yat-Sen University Cancer Center, Guangzhou, China; 5 Guangdong Provincial Key Laboratory of Malignant Tumor Epigenetics and Gene Regulation, Research Center of Medicine, Sun Yat-sen Memorial Hospital, Sun Yat-sen University, Guangzhou, China; 6 Department of Internal Medicine, Henan Cancer Hospital, Zhengzhou, China

**Keywords:** RPRD1B, Lymph node metastasis, Fatty acid metabolism, c-Jun/c-Fos/SREBP1 axis, NEAT1, hnRNPA2B1, TRIM25

## Abstract

**Background:**

Lymph node metastasis is one of most common determinants of the stage and prognosis of gastric cancer (GC). However, the key molecular events and mechanisms mediating lymph node metastasis remain elusive.

**Methods:**

RNA sequencing was used to identify driver genes responsible for lymph node metastasis in four cases of gastric primary tumors, metastatic lesions of lymph nodes and matched normal gastric epithelial tissue. qRT–PCR and IHC were applied to examine RPRD1B expression. Metastatic functions were evaluated in vitro and in vivo. RNA-seq was used to identify target genes. ChIP, EMSA and dual luciferase reporter assays were conducted to identify the binding sites of target genes. Co-IP, RIP, MeRIP, RNA-FISH and ubiquitin assays were applied to explore the underlying mechanisms.

**Results:**

The top 8 target genes (RPRD1B, MAP4K4, MCM2, TOPBP1, FRMD8, KBTBD2, ADAM10 and CXCR4) that were significantly upregulated in metastatic lymph nodes of individuals with GC were screened. The transcriptional cofactor RPRD1B (regulation of nuclear pre-mRNA domain containing 1B) was selected for further characterization. The clinical analysis showed that RPRD1B was significantly overexpressed in metastatic lymph nodes and associated with poor outcomes in patients with GC. The Mettl3-induced m^6^A modification was involved in the upregulation of RPRD1B. Functionally, RPRD1B promoted lymph node metastasis capabilities in vitro and in vivo. Mechanistic studies indicated that RPRD1B increased fatty acid uptake and synthesis by transcriptionally upregulating c-Jun/c-Fos and activating the c-Jun/c-Fos/SREBP1 axis. In addition, NEAT1 was upregulated significantly by c-Jun/c-Fos in RPRD1B-overexpressing cells. NEAT1, in turn, increased the stability of the RPRD1B mRNA by recruiting the m^6^A “reader” protein hnRNPA2B1 and reduced the degradation of the RPRD1B protein by inhibiting TRIM25-mediated ubiquitination. Notably, this functional circuitry was disrupted by an inhibitor of c-Jun/c-Fos/AP1 proteins (SR11302) and small interfering RNAs targeting NEAT1, leading to a preferential impairment of lymph node metastasis.

**Conclusions:**

Based on these findings, RPRD1B facilitated FA metabolism and assisted primary tumor implantation in lymph nodes via the c-Jun/c-Fos/SREBP1 axis, which was enhanced by a NEAT1-mediated positive feedback loop, serving as a potential therapeutic target for GC treatment.

**Supplementary Information:**

The online version contains supplementary material available at 10.1186/s13046-022-02449-4.

## Background

Gastric cancer (GC) is the fourth most common cancer and the second leading cause of cancer-related death worldwide [1]. As the chief culprit causing cancer-related death, metastasis is the ultimate challenge in our effort to fight cancer as a life-threatening disease [2]. Lymph node metastasis is closely related to tumor aggressiveness and a poorer prognosis for patients with GC. Based on accumulating evidence, lymph node metastasis is a source of cancer cells for further dissemination, suggesting that the mechanisms underlying lymph node metastasis are very important [3, 4]. However, most previous investigations of tumor metastasis have focused on the mechanism of distant metastasis rather than lymph node metastasis [5]. The molecular drivers leading to tumor lymph node metastasis remain obscure. Organ-specific metastasis in terms of organ tropism largely depends on the role of intrinsic cancer cell properties, such as driver genes and pathways regulating colonization [6], and the premetastatic microenvironment of specific organs [7].

We identified driver genes responsible for the lymph node metastasis of GC by applying RNA sequencing to analyze gene expression profiles in 4 cases of primary tumors, metastatic lesions of lymph nodes and their matched nontumor tissues. Among the differentially expressed genes, RPRD1B, a transcriptional coactivator, attracted our attention due to its dramatic upregulation in metastatic lymph nodes. RPRD1B, also named CREPT, encodes a nuclear protein that has been reported to be involved in promoting the transcription of cyclin D1 [8] and regulating DNA mismatch repair [9]. Upregulation of PRRD1B has been detected in various solid tumors, including colorectal cancer [10] and non-small cell lung cancer [11]. In gastric cancer, CREPT/RPRD1B has been reported to accelerate the G2/M transition mediated by Aurora kinase B and the ROS-related p53 pathway [12, 13]. However, the biological role of RPRD1B in GC metastasis is unknown.

Lipid biology was reported to be an essential process in lymph node metastasis, such as fatty acid (FA) metabolism, adipogenesis and cholesterol homeostasis [14]. Compared with the primary tumors, more significant accumulation of fatty acids was detected in lymph node metastatic tumors. In the lipid-rich lymph node, metastatic tumor cells preferred fatty acids rather than glucose as major fuel for energy production. In addition, transcriptional regulation is a crucial component involved in tumor metastasis and progression [15]. Transcriptional coactivators selectively promote lymph node metastatic tumors, leading to the activation of genes in the fatty acid metabolism signaling pathway. Yes-associated protein (YAP) is required for the metabolic shift towards fatty acid oxidation and the lymph node metastasis of tumors [14]. Transcription factor activator protein-1 (AP1), as a heterodimer composed of proteins from the Jun and Fos families, transcriptionally regulates numerous genes involved in fatty acid metabolism and nonalcoholic fatty liver disease (NAFLD) [16, 17]. Furthermore, long noncoding RNAs (lncRNAs) have been identified as master regulators of lipid metabolism [18]. The expression of nuclear enriched abundant transcript 1 (NEAT1) is strongly induced by transcription factors such as CCAAT/enhancer-binding protein α (CEBPα) and peroxisome proliferator-activated receptor γ (PPARγ) during adipogenesis [19].

In the present study, we proposed a novel mechanism by which RPRD1B overexpression promotes fatty acid metabolism via the c-Jun/c-Fos/SREBP1 axis that is enhanced by a NEAT1-mediated regenerative feedback loop, contributing to lymph node metastasis and the poor prognosis of advanced GCs.

## Methods

### Clinical specimens and cell lines

A cohort of 42 paired fresh specimens of tumor and adjacent non-tumor tissues, including 12 cases of matched metastatic lymph-node, were collected immediately following gastrectomy of GC patients at the FAHZZU (First Affiliated Hospital of Zhengzhou University, Zhengzhou, China). No patient in this study received preoperative treatment. Informed consent was obtained from all patients before the collection of gastric specimens and samples used in this study were approved by the Committees for Ethical Review of Research at Zhengzhou University. Immortalized gastric epithelial cell line GES1 and seven GC cell lines MKN28, MGC803, BGC823, HGC27, SGC7901, AGS and NCI-N87 were obtained from Chinese academy of Sciences (Shanghai, China). They were recently authenticated by STR profiling. Mycoplasma contamination was not found in these cell lines.

### Tissue microarray (TMA) and Immunohistochemical (IHC) staining

Another cohort with 300 cases of GC patients received radical surgery between 2010 and 2013 at FAHZZU were followed up in May of 2015. Integral survival information of 205 patients were obtained. The median follow-up was 38 months, ranged from 30 months to 60 months postoperatively. The follow-up period was started from the time of surgery to the time of death or telephone visiting. A TMA were constructed from the paraffin-embedded tissues of this cohort, containing 205 cases of primary GC specimens, paired adjacent non-tumor gastric tissues and 94 cases of matched metastatic lymph-node. IHC staining was performed using the standard streptavidin–biotin–peroxidase complex method as described previously [20]. Due to some inevitably causes, such as detachment of slice and target tissue absence, 191 cases of patients with integral staining were included in final statistical analysis. An immunoreactivity score system was applied in the analysis of IHC staining. The percentage of positive cells was scored as follows: 0, < 5%, 1, 5–25%, 2, 25–50%, 3, 50–75%, 4, 75–100%. The intensity of staining was scored as follows: 0, negative; 1, weak; 2, moderate; 3, strong. The total score was determined by the following formula: staining index = positive percentage × intensity. In present study, 191 cases of patients with assessable staining were included in statistical analysis. The optimum cut-off score was calculated by ROC curve analysis (IHC score ≥ 4 was defined as high expression, IHC score < 4 score was defined as low expression).

### Plasmids and reagents

The full-length RPRD1B, c-Jun, c-Fos, NEAT1_1 and Luciferase were cloned into expression vector plenti6(+) (Invitrogen, Carlsbad, CA), respectively. Stable transfection into HGC27 and SGC7901 cells using Lipofectamine 2000 (Invitrogen, Carlsbad, CA). Stable expressing clones were selected by blasticidin (Invitrogen, Carlsbad, CA). Lentiviral containing short hairpin RNAs (shRNA) targeting RPRD1B was purchased from GeneCopoeia (Rockville, MD) and transfected into AGS and BGC823 cells. Cells transfected with scramble vector were used as controls. Puromycin was used to select stable clones.

### Antibodies and Western blotting

Protein quantification assays were performed to determine the concentration for each collected lysate. Equal amounts of denatured protein were load into the wells of SDS-PAGE gel along with molecular weight marker and then transferred from gel to PVDF membrane (Millipore). After blocked with 5% non-fat milk, the membrane was incubated with primary antibody overnight at 4 °C. The membrane was washed with TBST, then incubated with secondary antibody 1 h at RT. Proteins were detected by enhanced chemiluminescence (ECL) system. The images were acquired using darkroom development techniques. Using antibodies listed in Supplementary Table 1.

### RNA extraction and quantitative real-time PCR

Total RNA was extracted using TRIZOL Reagent (Invitrogen), and then reverse transcription was performed using a PrimeScript™ RT Reagent Kit (Takara) with gDNA eraser. The cDNA was subjected to quantitative real-time PCR (qRT-PCR) using the SYBR Green PCR Kit (Roche) and the assay was performed on a Roche Lightcycler 480 Sequence Detector (Roche). Specificity of primers was verified by dissociation curve analysis. GAPDH was used as an internal control. All qRT-PCR reactions were performed in duplicates. Using primers listed in Supplementary Table 2.

### Migration and invasion assays

Migration and invasion assays were performed in 24-well milli-cell hanging insert (BD Biosciences) or 24-well BioCoat Matrigel Invasion Chambers (BD Biosciences). In brief, 5 × 10^4^ cells were seeded to the top chamber and 10% FBS in medium was added to the bottom chamber as a chemoattractant. After 24 or 48 h incubation, the number of cells that invaded through the membrane (migration) or Matrigel (invasion) was counted in 10 fields and imaged using SPOT imaging software (Nikon).

### Animals

All animal experiments were performed according to the guidelines of the Council on Animal Care and approved by Hongkong University. Briefly, 4–6 weeks old male BALB/c nude mice were used in present study. Lymph node metastasis animal model was established by injecting 2 × 10^5^ RPRD1B-overexpressed HGC27 and shRPRD1B-transfected AGS cells into right hind footpad of nude mice, respectively. Empty vector transfected cells were used as controls. Popliteal lymph nodes, which represent the sentinel lymph node for the model, were examined.

### RNA sequencing (RNA-seq) and gene set enrichment analysis (GSEA)

Total RNA was extracted using RNeasy Mini Kit (Qiagen) and measured for quality using Bioanalyzer (Agilent). RNA-seq libraries were enriched for stranded poly(A) mRNA and sequenced on Illumina HiSeq X Ten (Illumina). RNA sequencing was completed by Novogene Co. Ltd. (Beijing, China). KEGG and GO pathway analysis were performed on David (https://david.ncifcrf.gov/). The GSEA were performed using GSEA v2.0 software (http://www.broadinstitute.org/gsea).

### Luciferase reporter assay

Fragments encompassing the putative c-Jun/c-Fos promoter regions were inserted upstream of firefly luciferase (FLuc) coding sequences in the pGL3-basic reporter plasmid. HGC27-Lacz and HGC27-RPRD1B overexpression cells were seeded in 96-well plates at a density of 2 × 10^3^ cells per well respectively, and then co-transfected with pGL3-c-Jun/c-Fos promoter fragments and pTK-Renilla. FLuc and RLuc activities were determined after 48 h using the Dual-Luciferase Assay System (Promega, Madison, WI). Using primers listed in Supplementary Table 3.

### Chromatin Immunoprecipitation assay (CHIP)

All ChIP experiments were carried out using EZ-Magna ChIP kit ((Merck Millipore, Germany). Briefly, 1 × 10^7^ cells were prepared and then cross-linked with 1% formaldehyde for 10 min at room temperature (RT). The cross-linking process was quenched by adding 0.125 M glycine. And chromatin was isolated with lysis buffer provided in the kit. After that, sonication was performed to shear DNA to 200–1000 bp. Immunoprecipitation (IP) of cross-linked protein/DNA was done use 10 mg RPRD1B antibody (Abcam). Protein/DNA Complexes were eluted, and cross-links of protein/DNA were reversed with proteinase K. DNA was purified using spin column and used for PCR reactions. Fold enrichment was calculated based on CT as 2^-Δ(ΔCT)^, where ΔCt = CT_IP_ - CT_Input_ and Δ(ΔCT) = ΔCT_antibody_ -ΔCT_IgG_. Using primers listed in Supplementary Table 4.

### Electrophoretic mobility shift assay (EMSA)

Binding activity on the promoter region of RPRD1B (c-Jun-A, c-Jun-B, c-Jun-C, and c-Jun-D; c-Fos-A, c-Fos-B, c-Fos-C, and c-Fos-D, respectively) was detected by the EMSA Kit (Thermo Scientific, IL) according to the manufacturer’s protocol. Probes used in this study were synthesized by Invitrogen (China) and shown in Supplementary Table 5. Nuclear extracts were prepared using the NE-PER Nuclear and Cytoplasmic Extraction Reagents (Thermo Scientific). As negative controls, samples with biotin-c-Jun/c-Fos DNA but without nucleus protein were used. For competition experiments, 100-fold molar excess of unlabeled c-Jun/c-Fos DNA was added to the binding mixture 10 min before the addition of the labelled probe. Visualized bands were analyzed using a BioSens Gel Imaging System (BIOTOP, China).

### RNA-binding protein Immunoprecipitation assay (RIP)

All RIP experiments were performed using EZ-Magna RIP kit ((Merck Millipore, Germany). Briefly, 2–3 × 10^7^ cells were prepared for lysate. Magnetic beads-antibody complex for immunoprecipitation was prepared by using 10μg RPRD1B antibody (Abcam). Then, RNA-binding Protein-RNA complexes (RBP) were immunoprecipitated according to the kit protocol. Fold enrichment was calculated based on CT as 2^-Δ(ΔCT)^, where ΔCt = CT_IP_ - CT_Input_ and Δ(ΔCT) = ΔCT_antibody_ - ΔCT_IgG_. Using primers listed in Supplementary Table 3.

### RNA FISH for NEAT1 on cell lines

GC cells grown on coverslips were rinsed with phosphate-buffered saline (PBS) in DEPC and fixed with cold 4% paraformaldehyde for 5 min at RT. Subsequently, the cells were treated with Triton X-100 at a concentration of 0.3% for 30 min. NEAT1_1 probe was diluted by RNA FISH hybridization buffer (1:50) and incubated with cells overnight at 37 °C. Images were acquired using laser scanning confocal microscope ZEISS 510 (Germany).

### RNA pull-down assay

Biotinylated NEAT1_1 or antisense NEAT1_1 was constructed at Biosense Bioscience Co. Ltd. (Guangzhou, China), and then incubated with cellular protein extracts from HGC27-RPRD1B and SGC7901-RPRD1B cells, and streptavidin beads were then added. Recovered proteins associated with NEAT1_1 or antisense NEAT1_1 were resolved by gel electrophoresis.

### Methylated RNA Immunoprecipitation (MeRIP)

All RIP experiments were performed using Magna MeRIP m^6^A kit ((Merck Millipore, Germany). First, 2 × 10^7^ cells were prepared for total RNA extraction with TRIzol (Invitrogen) After fragmentation, RNA was incubated with magnetic beads-m^6^A antibody complex for immunoprecipitation. MeRIPed mRNA was then analyzed by qRT-PCR. A positive control methylated region (Human EEF1A positive) and a negative control unmethylated region (Human EEF1A1 negative) were used.

### Statistical analysis

SPSS 16.0 was used for all data analyses. A Pearson χ2 test was used for the correlation between clinicopathologic features and RPRD1B expression. Kaplan–Meier plots and log-rank tests were used for overall survival analysis. The independent Student t test was used for two-group comparisons. For comparisons among curves with different time points, the two-way ANOVA analysis were used. A *P* value less than .05 was considered statistically significant.

## Results

### RPRD1B is overexpressed in GC and metastatic lymph nodes

**Fig. 1:**
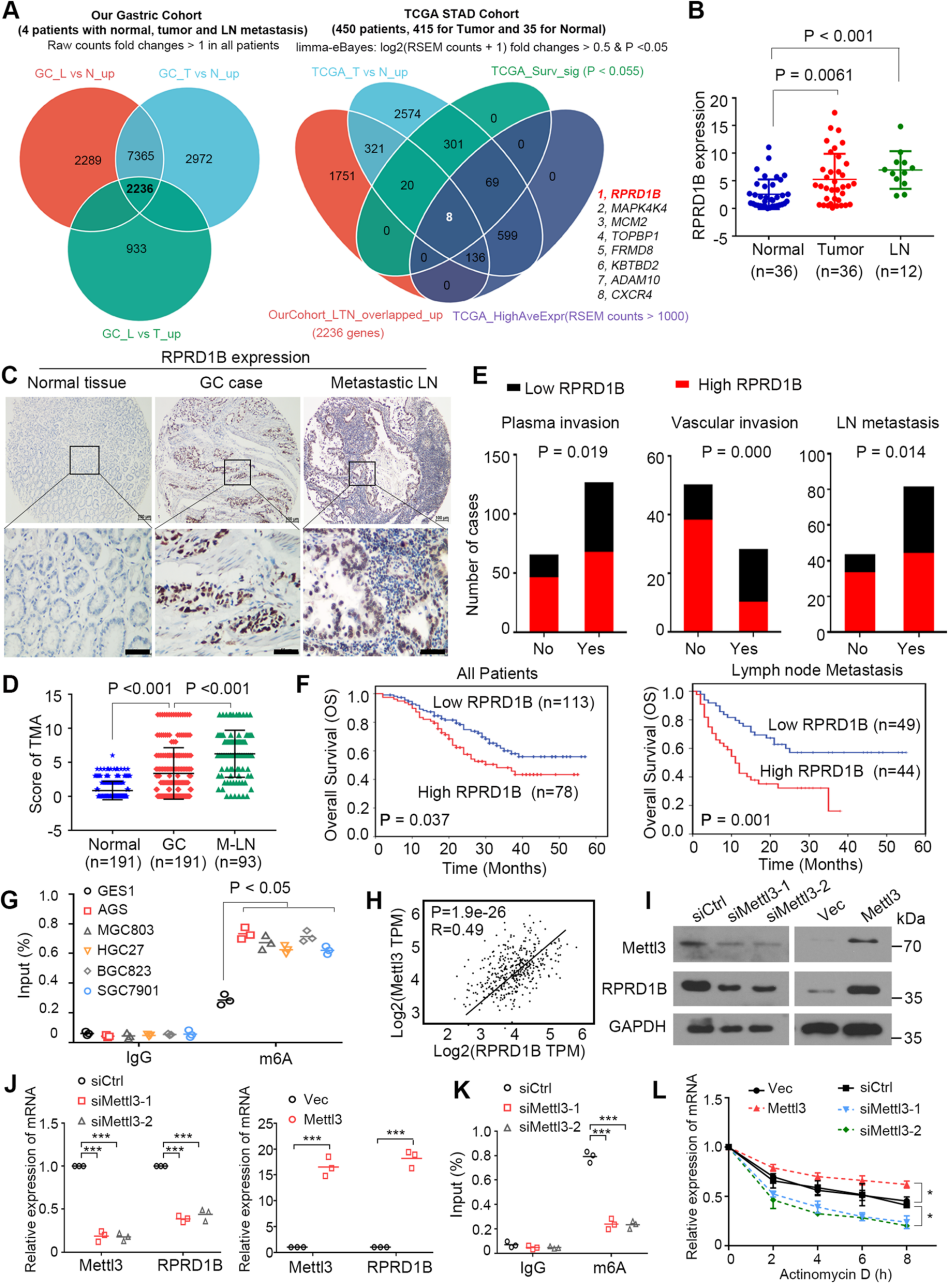
The m^6^A-induced upregulation of RPRD1B is involved in the lymph node metastasis of GCs. **A** Venn diagram showing eight genes putatively related to lymph node metastasis predicted by RNA sequencing of our GC cohort and TCGA database. **B** RT–qPCR showing that RPRD1B was overexpressed in both primary GC tissues (*n* = 36) and metastatic lymph nodes (*n* = 12) from our cohort. **C** Representative images of RPRD1B IHC staining of the TMA. Scale bar, 50 μm. **D** RPRD1B was overexpressed in both GC primary tumors (*n* = 191) and metastatic lymph nodes (*n* = 94) in the TMA. **E** Plasma invasion, vascular invasion and lymph node metastasis occurred more frequently in patients with high RPRD1B expression. **F** The Kaplan–Meier analysis revealed that high RPRD1B expression in tumors (*n* = 191) and metastatic lymph nodes (*n* = 93) was related to the shorter overall survival of patients with GC in our cohort. **G** RIP-qPCR analysis showing the stronger enrichment of m^6^A-modified RPRD1B in GC cells than in GES1 cells. **H** Positive correlation between RPRD1B expression and Mettl3 expression in TCGA data. **I**, **J** Levels of the RPRD1B and Mettl3 proteins and mRNAs after Mettl3 knockdown or overexpression in AGS cells. **K** RIP-qPCR showing the enrichment of m^6^A in AGS after Mettl3 depletion. **L** The decay rate of the RPRD1B mRNA after treatment with 2.5 μM actinomycin D for the indicated times in AGS cells with Mettl3 knockdown or overexpression. Data are presented as the means ± SD of three independent experiments. (*, *P* < 0.05; ***, *P* < 0.001)

High-throughput RNA-seq was performed in 4 patients in the Chinese gastric cancer cohort with primary tumors, metastatic lymph nodes and corresponding nontumor epithelial tissue (Supplementary RNA-seq Data 1). Overexpressed genes in lymph node metastasis compared with matched normal/primary tumors were analysed (fold changes of RSEM counts > 1), and 2236 target genes were selected for subsequent analysis. Compared with genes that were overexpressed and associated with shorter survival in the TCGA STAD cohort (450 patients, 415 for tumor tissues and 35 for normal tissues; limma-eBayes: log2(RSEM counts + 1) fold changes > 0.5 and *P* < 0.05), 8 target genes (RPRD1B, MAP4K4, MCM2, TOPBP1, FRMD8, KBTBD2, ADAM10 and CXCR4) were screened from these 2236 genes (Fig. 1A). The expression levels of the 8 target genes were examined by quantitative real-time PCR (qPCR) in 10 randomly selected GC specimens (Supplementary Fig. 1A). Due to the significant up-regulation in lymph node metastatic lesions in GC specimens, RPRD1B was selected for further study. The expression status of RPRD1B was then confirmed using qRT–PCR in 36 patients with GC. RPRD1B was upregulated in 20/36 (55.6%) GC primary tumor tissues (*P* = 0.0061) and 9/12 (75%) metastatic lymph nodes (*P* < 0.0001) compared with the corresponding nontumor tissues (Fig. 1B).

For the subsequent survival analysis, IHC staining was performed on a tissue microarray (Fig. 1C). Overexpression of RPRD1B (IHC score ≥ 4) was detected in 78/191 (40.8%) informative GC primary tumor tissues (*P* < 0.0001) and 44/93 (47.3%) metastatic lymph nodes (*P* < 0.0001) (Fig. 1D). Furthermore, RPRD1B overexpression was significantly associated with the tumor invasion depth (*P* = 0.019), lymph node invasion (*P* = 0.014), and vascular invasion (*P* = 0.000) (Fig. 1E) (Supplementary Table 6). The Kaplan–Meier analysis revealed that RPRD1B overexpression in tumors (*P* = 0.037) and lymph nodes (*P* = 0.001) was significantly associated with shorter overall survival (Fig. 1F).

### The Mettl3-induced m^6^A modification is involved in the upregulation of RPRD1B

The mechanism underlying the aberrant expression of RPRD1B is unknown. Previous studies have reported that the m^6^A modification modulates mRNA stability and plays a critical role in GC metastasis [21]. m^6^A RNA immunoprecipitation (RIP) revealed that the m^6^A modification of RPRD1B was significantly more enriched in GC cells than in normal GES1 cells (Fig. 1G). As Mettl3 is the key m^6^A methyltransferase, we evaluated Mettl3 expression and the correlation between Mettl3 and RPRD1B expression in TCGA database. Notably, Mettl3 was significantly upregulated in GC tissues, and Mettl3 expression was positively correlated with RPRD1B expression (R = 0.49, *P* < 0.05) (Fig. 1H). Additionally, Mettl3 depletion apparently reduced RPRD1B mRNA and protein levels. In contrast, Mettl3 overexpression increased the RPRD1B mRNA and protein levels in AGS and HGC27 cells (Fig. 1I, J, Supplementary Fig. 1B, C). MeRIP assays also revealed that Mettl3 silencing reduced the m^6^A modification of the RPRD1B mRNA in AGS and HGC27 cells (Fig. 1K, Supplementary Fig. 1D). Furthermore, we examined the stability of the RPRD1B mRNA upon Mettl3 overexpression and silencing in AGS cells. Actinomycin D was used to block de novo mRNA synthesis in cells, and the stability of the RPRD1B mRNA decreased upon Mettl3 depletion but increased with Mettl3 overexpression in AGS and HGC27 cells (Fig. 1L, Supplementary Fig. 1E). Thus, the m^6^A writer Mettl3 upregulates RPRD1B mRNA expression by promoting the m^6^A modification in GC cells.

### RPRD1B promotes metastasis of GC cells in vivo and in vitro

**Fig. 2:**
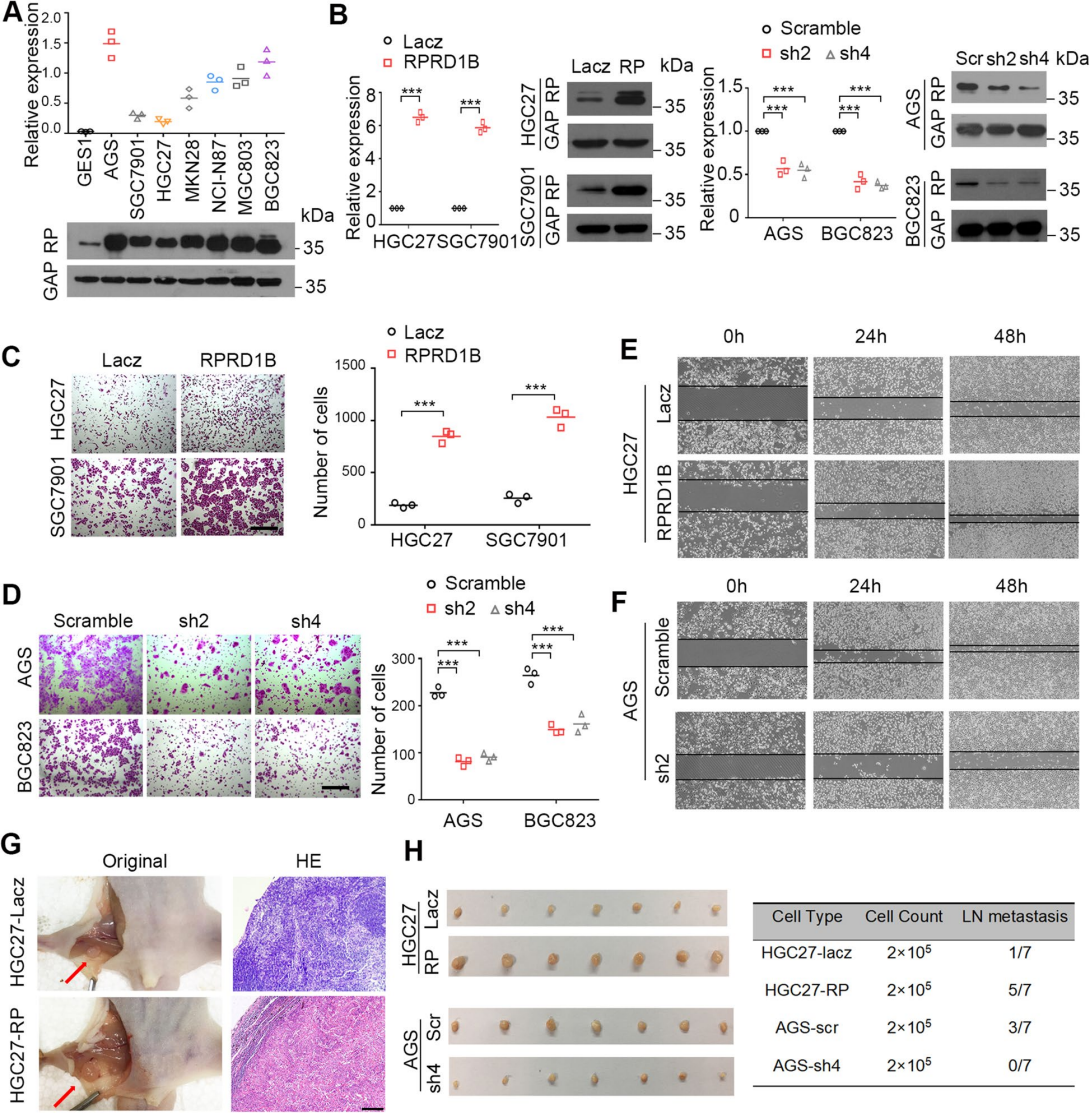
RPRD1B increases cell migration and tumor lymph node metastasis. **A** Upregulation of RPRD1B was detected in seven GC cell lines compared with an immortalized gastric cell line (GES1) using qRT–PCR and western blotting. **B** qRT–PCR and western blot analyses showing the ectopic expression of RPRD1B in RPRD1B-overexpressing cells and decreased expression of RPRD1B in shRNA-transfected cells. GAPDH expression was used as a loading control (**C**) Transwell assay showing that RPRD1B promoted cell migration and invasion. Scale bar, 200 μm. **D** Silencing RPRD1B expression effectively inhibited cell migration and invasion, as analyzed using the Transwell assay. Scale bar, 200 μm. **E**, **F** Wound-healing assay showing that RPRD1B overexpression promoted the migration of HGC27 cells and RPRD1B knockdown inhibited the migration of AGS cells at 0, 24, and 48 h after scratch wounding. **G** An in vivo lymph node metastasis assay was performed to evaluate the effect of LacZ- and RPRD1B-transfected cells and Scr- and shRPRD1B-transfected AGS cells on tumor metastasis (left panel). Representative images of H&E-stained lymph node metastases after the footpad injection of the indicated cells are shown (right panel). Scale bar, 200 μm. **H** Representative lymph node metastases formed in nude mice injected with the indicated cells. The number of metastatic lymph nodes is summarized (*n* = 7 mice/group). Data are presented as the means ± SD of three independent experiments. (***, *P* < 0.001)

Since the clinicopathological analysis revealed that RPRD1B overexpression was associated with vascular invasion and lymph node metastasis, we next investigated the effect of RPRD1B on tumor cell migration and invasion. Overexpression of RPRD1B was detected in 7 GC cell lines compared with the immortalized gastric cell line GES1 using western blotting and qRT–PCR (Fig. 2A). RPRD1B was stably overexpressed in HGC27 and SGC7901 cells and ablated with two short hairpin RNAs (shRNAs) in AGS and BGC823 cells (Fig. 2B). In vitro, RPRD1B overexpression potentiated the ability of HGC27 and SGC7901 cells to migrate and invade (Fig. 2C). Conversely, RPRD1B depletion in AGS and BGC823 cells resulted in the opposite effects (Fig. 2D). The wound-healing assay showed faster closure of the scratched “wound” by RPRD1B-overexpressing cells but slower closure by RPRD1B-silenced cells than in control cells (Fig. 2E, F, Supplementary Fig. 2A, B).

An animal model of lymph node metastasis was established to test the metastasis-promoting effect of RPRD1B in vivo. Swollen popliteal lymph nodes were observed in all seven mice injected with RPRD1B-overexpressing HGC27 cells, and lymph node metastasis was confirmed by H&E staining in 5/7 mice (Fig. 2G). Only 1/7 of mice injected with control cells displayed tumor metastasis. In contrast, after RPRD1B was silenced in AGS cells, none of the mice exhibited popliteal lymph node metastasis compared with 3/7 mice exhibiting metastasis after the injection of control cells (Fig. 2H).

### RPRD1B facilitates fatty acid metabolism by activating the c-Jun/c-Fos/SREBP1 axis

**Fig. 3:**
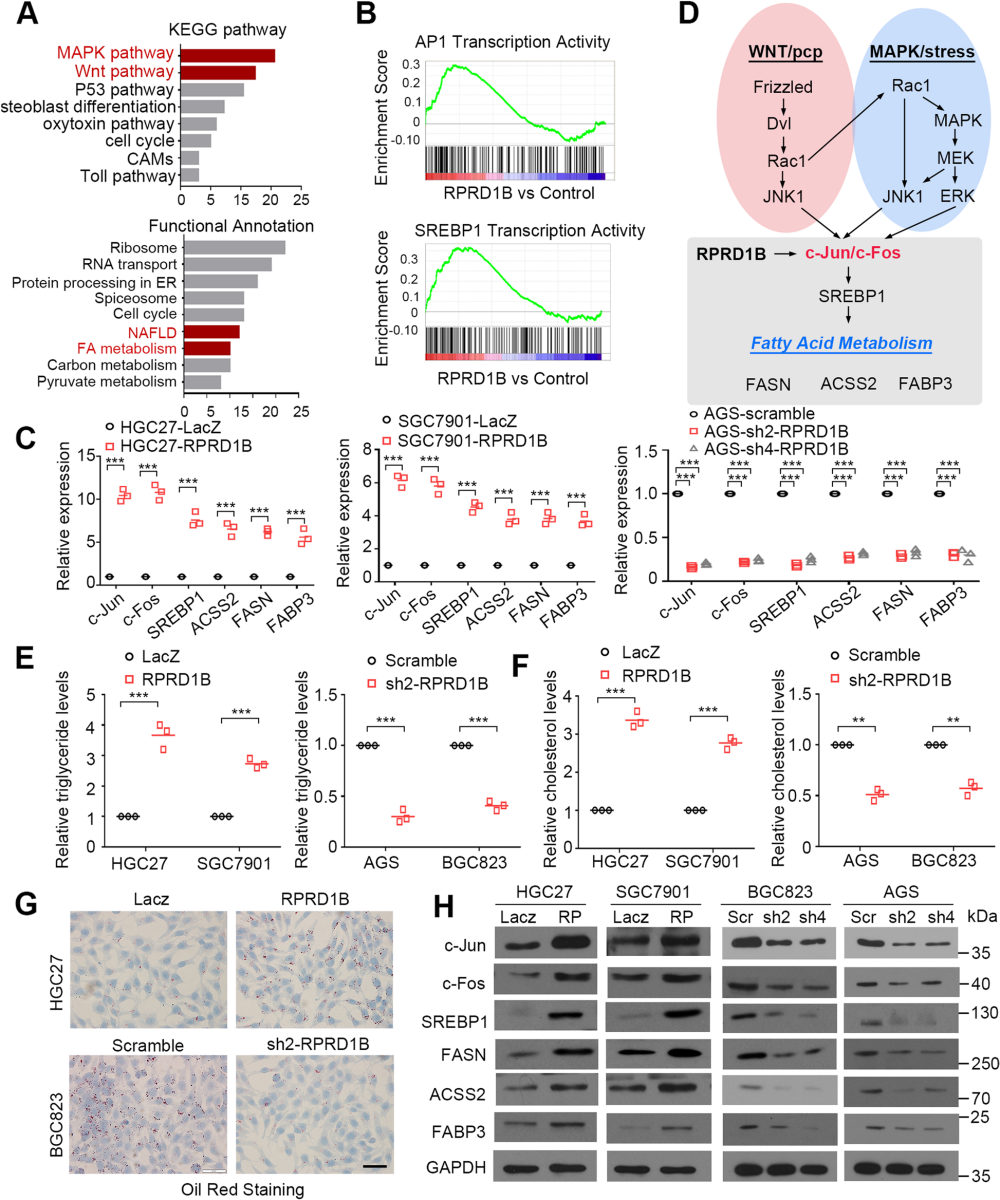
RPRD1B facilitates fatty acid uptake and synthesis by activating the c-Jun/c-Fos/SREBP1 axis. **A** The KEGG pathways and functional annotations were analyzed from a transcriptome analysis identifying genes that are upregulated upon RPRD1B overexpression. Enriched terms involved in RPRD1B function were the MAPK pathway, Wnt pathway, and fatty acid metabolism. **B** Representative GSEA plots were enriched in AP1 and SREBP1 transcriptional activity upon RPRD1B overexpression. **C** Relative mRNA levels of fatty acid metabolism-related genes were examined in LacZ- and RPRD1B-, Scr- and shRPRD1B-stably transfected cells as determined using qRT–PCR. **D** A schematic diagram of pathways identified in the KEGG pathway analysis and functional annotations. RPRD1B activated fatty acid metabolism through the c-Jun/c-Fos/SREBP1 pathway. **E**, **F** Cellular cholesterol and triglyceride levels were assessed in GC cells with RPRD1B knockdown or overexpression. **G** Oil Red O staining showing lipid droplets in GC cells with RPRD1B knockdown or overexpression. Scale bar, 50 μm. **H** Levels of the RPRD1B, c-Jun, c-Fos, SREBP1, FASN, ACSS2 and FABP3 proteins were determined in LacZ- and RPRD1B-, Scr- and shRPRD1B-stably transfected cells. GAPDH served as a loading control. Data are presented as the means ± SD of three independent experiments. (**, *P* < 0.01; ***, *P* < 0.001)

RNA sequencing analysis was applied between RPRD1B- and LacZ-transfected HGC27 cells to identify the differentially expressed genes regulated by RPRD1B. Ninety differentially expressed genes were identified (qval < 0.05, Supplementary RNA-seq Data 2). KEGG pathway analysis showed enrichment in the Wnt/PCP and mitogen-activated protein kinase (MAPK) signaling pathways. Functional annotation revealed that RPRD1B was involved in fatty acid (FA) metabolism and NAFLD, serving as a transcription cofactor binding to DNA fragments (Fig. 3A). GSEA indicated the significant enrichment of AP1 and SREBP1 transcriptional activity in RPRD1B-overexpressing cells (Fig. 3B).

Quantitative RT–PCR analysis was conducted to confirm the differentially expressed genes in RNA sequencing analysis. As the intersection of Wnt and MAPK pathway, the transcription factors c-Jun and c-Fos were most significantly upregulated. In addition to the transcription factors c-Jun and c-Fos, the fatty acid metabolism-related genes SREBP1, FASN, ACSS2 and FABP3 were upregulated in RPRD1B-overexpressing cells. An opposite expression pattern of these genes was observed in RPRD1B-silenced cells (Fig. 3C). As SREBP1 has a known role in regulating FA synthesis [22, 23] and was reported to be the target of AP1 members such as c-Jun and JNK2 [24, 25], we hypothesized that RPRD1B promotes fatty acid metabolism and lymph node metastasis by activating c-Jun/c-Fos/SREBP1 axis and increasing FA uptake and synthesis (Fig. 3D). Cholesterol and triglyceride levels were increased in RPRD1B-overexpressing cells but decreased in RPRD1B knockdown cells (Fig. 3E, F). Oil Red O staining was performed to verify the presence of more droplets in RPRD1B-overexpressing cells and fewer droplets in RPRD1B-knockdown cells (Fig. 3G). Positive correlations between c-Jun, c-Fos, SREBP1, FASN, ACSS2, FABP3 and RPRD1B expression at the protein level were examined using western blot analysis (Fig. 3H).

### RPRD1B occupies the promoter of c-Jun and c-Fos

**Fig. 4:**
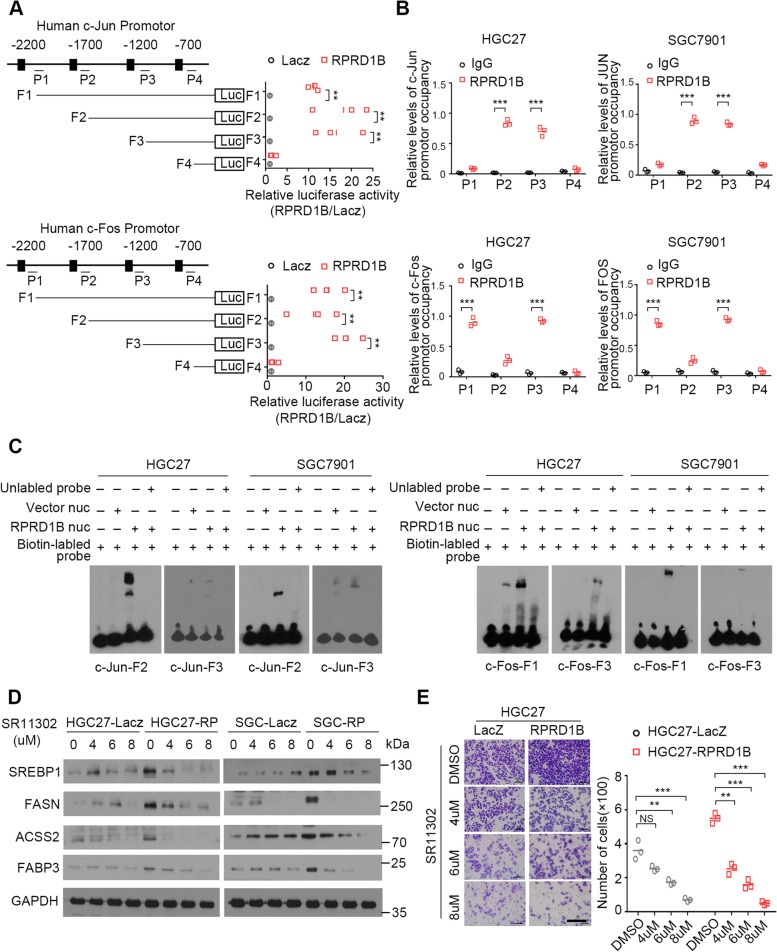
RPRD1B occupies the promoters of c-Jun and c-Fos. **A** Luciferase activity assays showed that RPRD1B significantly increased both c-Jun and c-Fos transcriptional activity. **B** ChIP assay showing that the c-Jun and c-Fos promoters were enriched in RPRD1B-ChIPed DNA fragments but not in IgG-ChIPed controls. **C** An electrophoretic mobility shift assay was performed to detect the interaction between RPRD1B and c-Jun (left panel) and between RPRD1B and c-Fos (right panel) double-stranded DNA probes. **D** western blot analysis showing that the AP1 inhibitor SR11302 effectively decreased the expression of SREBP1, FASN, ACSS2 and FABP3 induced by RPRD1B. **E** Transwell migration assay showing that SR11302 inhibited the RPRD1B-induced migration of HGC27 cells. Scale bar, 200 μm. Data are presented as the means ± SD of three independent experiments. (NS, not significant; **, *P* < 0.01; ***, *P* < 0.001)

We further examined the mechanism by which RPRD1B upregulates the expression of c-Jun and c-Fos using bioinformatics software (UCSC, PROMO and TFSEARCH) to analyse a 2 kb region upstream of the transcription start sites of c-Jun and c-Fos. All potential binding sites in the c-Jun and c-Fos promoters were predicted. A luciferase activity assay was then performed with four fragments of c-Jun or c-Fos promoter regions that encompass the predicted RPRD1B binding sites (F1: − 2200 bp to + 100 bp; F2: − 1700 bp to + 100 bp; F3: − 1200 bp to + 100 bp; F4: − 700 bp to + 100 bp). RPRD1B could significantly increase the transcriptional activity of both c-Jun and c-Fos in cells transfected with the F1, F2 and F3 fragments (*P* < 0.01) (Fig. 4A). A ChIP-PCR assay was then completed using four pairs of primers according to the predicted binding sites within the c-Jun promoter (P1: − 2157 bp to − 2002 bp, P2: − 1626 bp to − 1471 bp, P3: − 1194 bp to − 1049 bp, and P4: − 690 bp to − 535 bp) and c-Fos promoter (P1: − 2135 bp to − 2010 bp, P2: − 1622 bp to − 1517 bp, P3: − 1160 bp to − 1025 bp and P4: − 633-bp to 508 bp). The P2 and P3 sequences in the c-Jun promoter and the P1 and P3 sequences in the c-Fos promoter were enriched in RPRD1B-ChIPed DNA fragments but not in IgG-ChIPed controls (*P* < 0.001) (Fig. 4B). Electrophoretic mobility shift assays (EMSAs) were performed to further confirm that more probes of c-Jun and c-Fos were pulled down in RPRD1B-overexpressing cells (Fig. 4C).

We inhibited the expression of c-Jun/c-Fos by applying SR11302 (AP1 inhibitor) to confirm that RPRD1B promotes tumor metastasis by activating the c-Jun/c-Fos/SREBP1 pathway. SR11302 effectively inhibited the expression of SREBP1, FASN, ACSS2 and FABP3 in a dose-dependent manner (Fig. 4D). Cell migration assays indicated that SR11302 significantly decreased cell motility in RPRD1B-overexpressing HGC27 and SGC7901 cells (Fig. 4E, Supplementary Fig. 2C).

### The lncRNA NEAT1 is transcriptionally upregulated by c-Jun/c-Fos/AP1

**Fig. 5:**
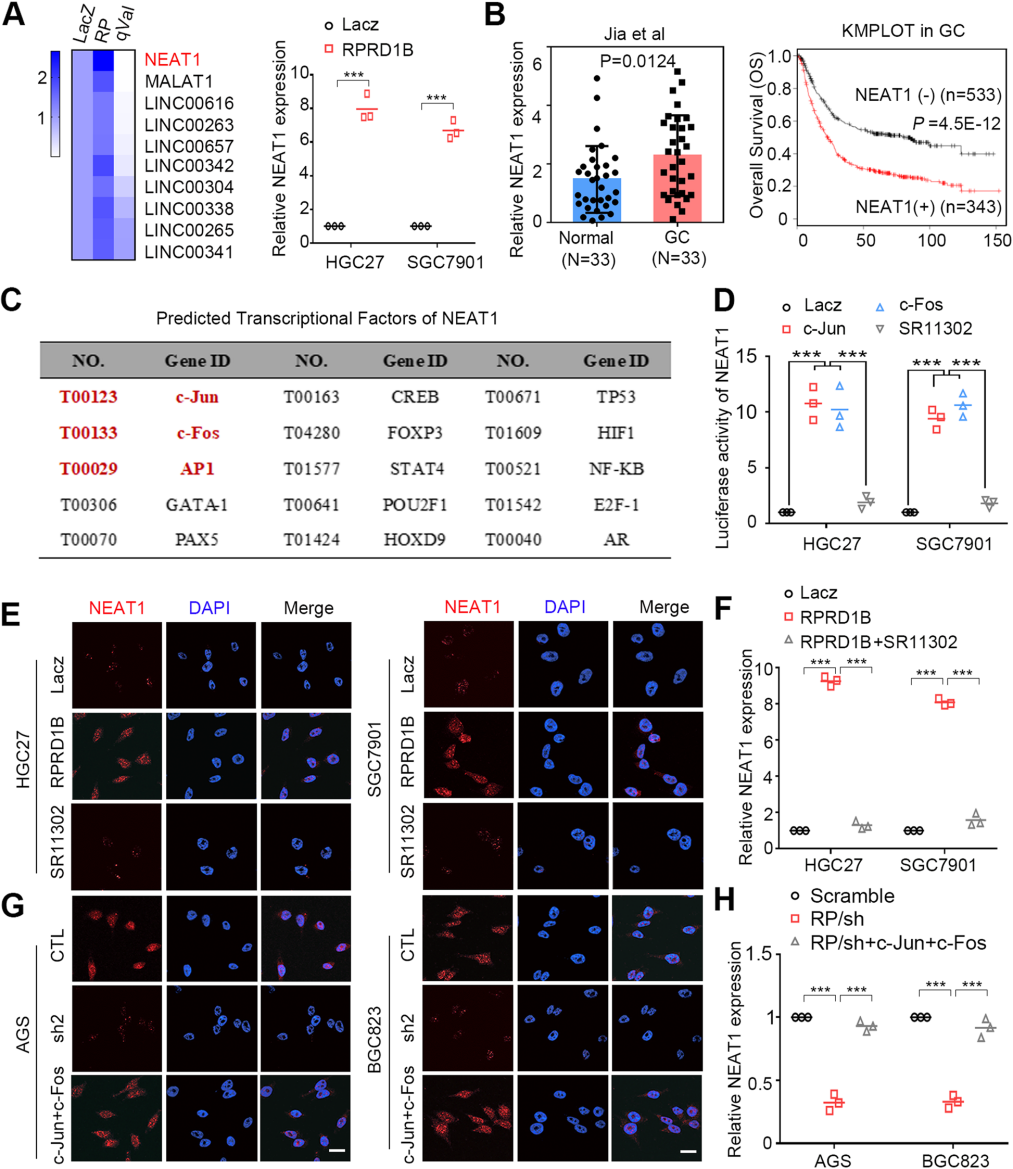
c-Jun and c-Fos upregulate NEAT1 transcription. **A** Heatmap showing the list of upregulated lncRNAs in the RNA sequencing data between LacZ- and RPRD1B-overexpressed HGC27 cells, according to q value (qualified *P* value). NEAT1 ranked first with the most significance (q = 9.38E-49). The mRNA expression level of NEAT1 were confirmed upregulation in RPRD1B-overexpressing HGC27 and SGC7901 cells. **B** NEAT1 overexpression was confirmed in our cohort of patients with GC (*n* = 33). Overall survival curves for patients with GC in TCGA cohort according to the expression status of NEAT1 (*n* = 876). **C** Potential transcription factors of NEAT1 were predicted in ALGGEN website. First 15 transcriptional factors including c-Jun, c-Fos and AP1 were listed. **D** The luciferase activity of NEAT1 was increased following c-Jun/c-Fos transfection in HGC27 and SGC7901 cells. The effect was diminished by SR11302. **E**, **F** RNA-FISH and qRT–PCR showed that NEAT1 was upregulated in RPRD1B-overexpressing HGC27 and SGC7901 cells. The effect was diminished by SR11302. **G**, **H** NEAT1 was downregulated in AGS and BGC823 RPRD1B-silenced cells and rescued by c-Jun and c-Fos. Scale bar, 20 μm. Data are presented as the means ± SD of three independent experiments. (***, *P* < 0.001)

According to previous studies, lncRNAs are key molecules that regulate transcriptional and posttranscriptional processes [26, 27]. In the present study, we noticed that the lncRNA NEAT1 was significantly upregulated in the RNA-seq data conducting between LacZ- and RPRD1B-overexpressing cells (Fig. 5A). In clinical samples, NEAT1 was frequently upregulated and significantly correlated with shorter survival of patients with GC in our cohort and TCGA database (Fig. 5B). However, the mechanism of NEAT1 upregulation in RPRD1B-overexpressing GC cells is unknown. The potential transcription factor and promoter region involved in NEAT1 transcription were analyzed by ALGGEN and Promoter 2.0 to further identify the mechanism of NEAT1 upregulation. Interestingly, c-Jun/c-Fos/AP1 were predicted to be transcription factors for NEAT1 (Fig. 5C). We validated this prediction by performing a luciferase activity assay with the promoter DNA fragments of NEAT1 containing binding sites for c-Jun and c-Fos. NEAT1 transcriptional activity was significantly upregulated following c-Jun and c-Fos transfection. The effect was significantly diminished by SR11302 (Fig. 5D). RNA-FISH and qRT-PCR confirmed the upregulation of NEAT1 in RPRD1B-upregulated GC cells, which was decreased significantly by SR11302 (Fig. 5E, F). The opposite effect was detected in RPRD1B-silenced GC cells (Fig. 5G, H). Transwell assays verified that the invasive ability of RPRD1B was blocked by SR11032 in RPRD1B-upregulated cells, and rescued by overexpressing c-Jun/c-Fos in RPRD1B-silenced GC cells (Supplementary Fig. 2D, E). These data suggested that RPRD1B transcriptionally upregulates NEAT1 in a c-Jun/c-Fos/AP1-dependent manner.

### NEAT1 increases RPRD1B mRNA stability via the hnRNPA2B1-mediated m^6^A modification

**Fig. 6:**
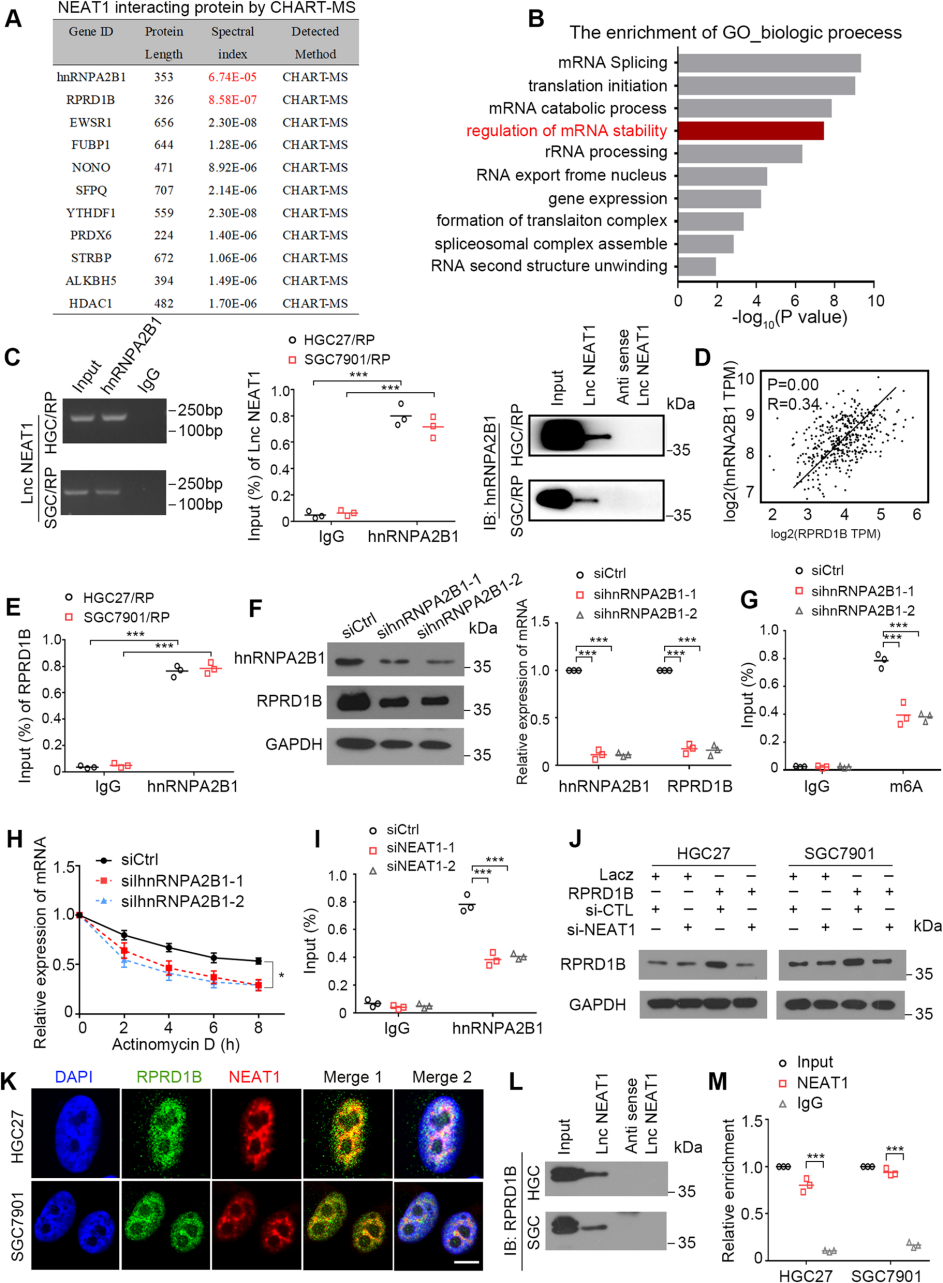
NEAT1 increases the mRNA stability of RPRD1B by recruiting hnRNPA2B1. **A** The datasheet shows part of NEAT1-interacting proteins obtained from CHART-MS. **B** GO analysis showed that the functions of NEAT1-interacting proteins were mostly enriched in RNA processing. **C** RIP assays show that NEAT1 is pulled down by hnRNPA2B1 antibody in RPRD1B-overexpressing HGC27 and SGC7901 cells. Immunoprecipitation with control IgG served as the negative control. (left and middle panels). Pull-down assays showed that hnRNPA2B1 was pulled down by NEAT1. Antisense of NEAT1 was used as negative control (right panel). **D** Positive correlation between RPRD1B expression and hnRNPA2B1 expression in TGCA data. **E** RIP-qPCR showing the enrichment of hnRNPA2B1 on the RPRD1B mRNA in RPRD1B-overexpressing HGC27 and SGC7901 cells. **F** Levels of the hnRNPA2B1 and RPRD1B proteins and mRNAs after RPRD1B inhibition in RPRD1B-overexpressing HGC27 cells. The results are summarized as the means ± SD of three independent experiments. **G** MeRIP-qPCR showing the enrichment of m^6^A in HGC27 cells after hnRNPA2B1 depletion. **H** The decay rate of the RPRD1B mRNA after treatment with 2.5 μM actinomycin D for the indicated times following hnRNPA2B1 knockdown in RPRD1B-overexpressing HGC27 cells. **I** RIP-qPCR showing the enrichment of hnRNPA2B1 on the RPRD1B mRNA in RPRD1B-overexpressing HGC27 cells with NEAT1 silencing. **J** The expression of RPRD1B was reduced following siNEAT1 transfection. **K** Representative IF staining images of the colocalization of NEAT1 and RPRD1B in RPRD1B-transfected HGC27 and SGC7901 cells. Scale bar, 20 μm. **L**, **M** Pull-down and RIP assay showing that NEAT1 interacted with RPRD1B in RPRD1B-transfected HGC27 and SGC7901 cells. Antisense of NEAT1 was used as negative control in pull-down assay. Immunoprecipitation with control IgG served as the negative control in RIP assay. GAPDH was served as the loading control. Data are presented as the means ± SD of three independent experiments. (*, *P* < 0.05, ***, *P* < 0.001)

Nuclear speckles and paraspeckles have been reported to be important locations involved in posttranscriptional processes, such as splicing of pre-mRNA and m^6^A methylation of mRNA [28]. As an essential component of nuclear paraspeckles, NEAT1 was reported to contribute to a cancer-favorable transcriptional program [29]. We explored whether NEAT1 is a transcriptional or posttranscriptional modifier of RPRD1B by identifying the proteins interacting with NEAT1 through capture hybridization analysis of RNA targets and mass spectrometry (CHART/MS) performed with a probe against NEAT1, as described in a recent study [30]. Interestingly, RNA-binding proteins involved in m^6^A modification, such as hnRNPA2B1, YTHDF1 and hnRNPC, were precipitated in protein complexes pulled down by NEAT1 (Fig. 6A). Gene Ontology (GO) analysis of NEAT1-interacting genes showed enrichment in several biological processes, such as mRNA splicing, translation initiation and mRNA stability (Fig. 6B). As one of the predicted proteins, the interaction of NEAT1 and hnRNPA2B1 was confirmed by RNA pull-down and RIP experiments (Fig. 6C, Supplementary Fig. 3A). Notably, hnRNPA2B1, a well-known m^6^A reader, was reported to affect the posttranscriptional modification of m^6^A-methylated RNA in the nucleus, such as splicing, stability and translation. However, researchers had not determined whether NEAT1/hnRNPA2B1 participates in the posttranscriptional regulation of m^6^A-methylated RPRD1B. Positive correlations between RPRD1B and hnRNPA2B1 expression at the mRNA level were confirmed in TCGA database (R = 0.54, *P* = 0.00) (Fig. 6D). RIP assays confirmed the direct interaction between hnRNPA2B1 and the RPRD1B mRNA in HGC27 and SGC7901 cells (Fig. 6E). Silencing of hnRNPA2B1 in RPRD1B-overexpressing HGC27 and SGC7901 cells significantly decreased the RPRD1B mRNA and protein levels (Fig. 6F, Supplementary Fig. 3B). MeRIP assays revealed that silencing hnRNPA2B1 reduced m^6^A modification of RPRD1B mRNA in HGC27 and SGC7901 cells (Fig. 6G, Supplementary Fig. 3C). After actinomycin D treatment, the half-life of the RPRD1B mRNA was significantly reduced due to hnRNPA2B1 depletion in HGC27 and SGC7901 cells (Fig. 6H, Supplementary Fig. 3D). In addition, the interaction between hnRNPA2B1 and the RPRD1B mRNA was impaired after NEAT1 suppression in HGC27 and SGC7901 cells (Fig. 6I, Supplementary Fig. 3E). Moreover, RPRD1B was downregulated after NEAT1 silencing in HGC27 and SGC7901 cells (Fig. 6J). Interestingly, we found that RPRD1B was also present in the protein complex precipitated by the *NEAT1* probe, suggesting a direct interaction between NEAT1 and the RPRD1B protein. Immunofluorescence (IF) staining for RPRD1B and RNA-FISH of NEAT1 were performed to verify their colocalization in RPRD1B-transfected HGC27 and SGC7901 cells (Fig. 6K). Their interaction was then confirmed by performing RNA pull-down and RIP experiments (Fig. 6L, M). Taken together, our data suggest that NEAT1 facilitates hnRNPA2B1 binding to the RPRD1B mRNA and increases its stability in an m^6^A-dependent manner.

### NEAT1 reduces degradation of RPRD1B by inhibiting TRIM25-mediated ubiquitination

**Fig. 7:**
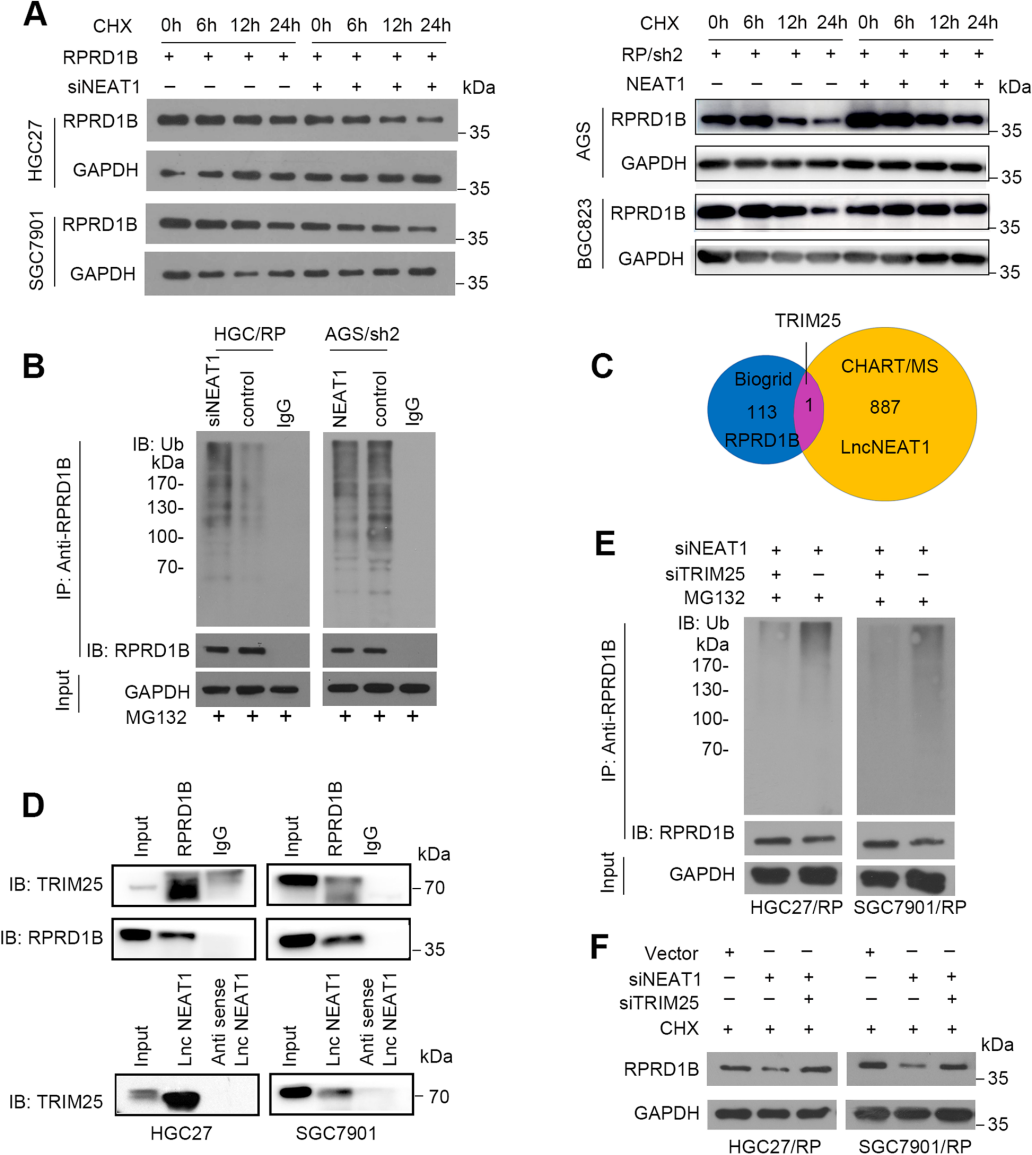
NEAT1 reduces TRIM25-mediated ubiquitination of RPRD1B. **A** CHX assays showed that overexpression of NEAT1 reduced the degradation of RPRD1B, in opposite, the silencing of NEAT1 accelerated the degradation of RPRD1B. GAPDH was used as a loading control. **B** MG132-induced accumulation of polyubiquitinated RPRD1B was decreased in NEAT1-overexpressed cells, and increased in NEAT1-silenced cells compared with control cells. GAPDH was used as a loading control. **C** Venn diagram showing that TRIM25 was the single overlapping protein between the set of proteins that interacted with RPRD1B and NEAT1. **D** Co-IP and pull-down assays confirmed the interaction between RPRD1B and TRIM25 and between NEAT1 and TRIM25. **E** After MG132 treatment, polyubiquitinated RPRD1B accumulation was induced by siNEAT1 but restrained by TRIM25 knockdown. **F** The CHX assay verified that RPRD1B degradation was enhanced by the silencing of NEAT1 but rescued by TRIM25 knockdown in GC cells. GAPDH was served as the loading control

Emerging studies reported that RNA binding protein (RBP) participated in R-loops formation and played important roles in genome stability and gene expression [31, 32]. RPRD1B was predicted to function as a new potential RBP for involving in R-loops formation [33]. However, the manner of interaction and modification between RPRD1B and NEAT1 was unknown. Ubiquitin assays were performed to explore the mechanism between NEAT1 and RPRD1B protein. Cells with NEAT1 silencing were then treated with the protein synthesis inhibitor cycloheximide, and a noticeably shorter half-life of RPRD1B was detected in HGC27 and SGC7901 cells than in control cells. In contrast, NEAT1 overexpression extended the half-life of RPRD1B in AGS and BGC823 cells (Fig. 7A). Based on these results, the NEAT1-mediated ubiquitin–proteasome pathway may be involved in the stabilization of the RPRD1B protein. The proteasome inhibitor MG132 was used to verify whether NEAT1 inhibited the degradation of RPRD1B. Increased levels of polyubiquitinated RPRD1B were observed in NEAT1-silenced cells compared with control cells. In contrast, reduced accumulation of polyubiquitinated RPRD1B was detected in NEAT1-overexpressing cells compared with control cells (Fig. 7B). We surveyed a protein–protein interaction database, Biogrid (www.biogrid.org), to identify the mechanism of RPRD1B ubiquitination and identified 113 RPRD1B interactors, including the E3 ubiquitin ligase TRIM25. As an RNA-binding protein, TRIM25 also interacted with NEAT1 in CHART-MS (Fig. 7C). IP assays confirmed the interaction among RPRD1B, NEAT1 and TRIM25 (Fig. 7D), verifying our hypothesis. NEAT1-silencing mediated RPRD1B ubiquitination was rescued by TRIM25 knockdown in GC cells (Fig. 7E). RPRD1B degradation was rescued by TRIM25 knockdown in NEAT1-silenced GC cells (Fig. 7F). According to these results, we concluded that NEAT1 decreases RPRD1B ubiquitination by blocking its interaction with TRIM25.

### The RPRD1B/c-Jun/c-Fos/SREBP1 axis correlates with the lymph node metastasis of GC

**Fig. 8:**
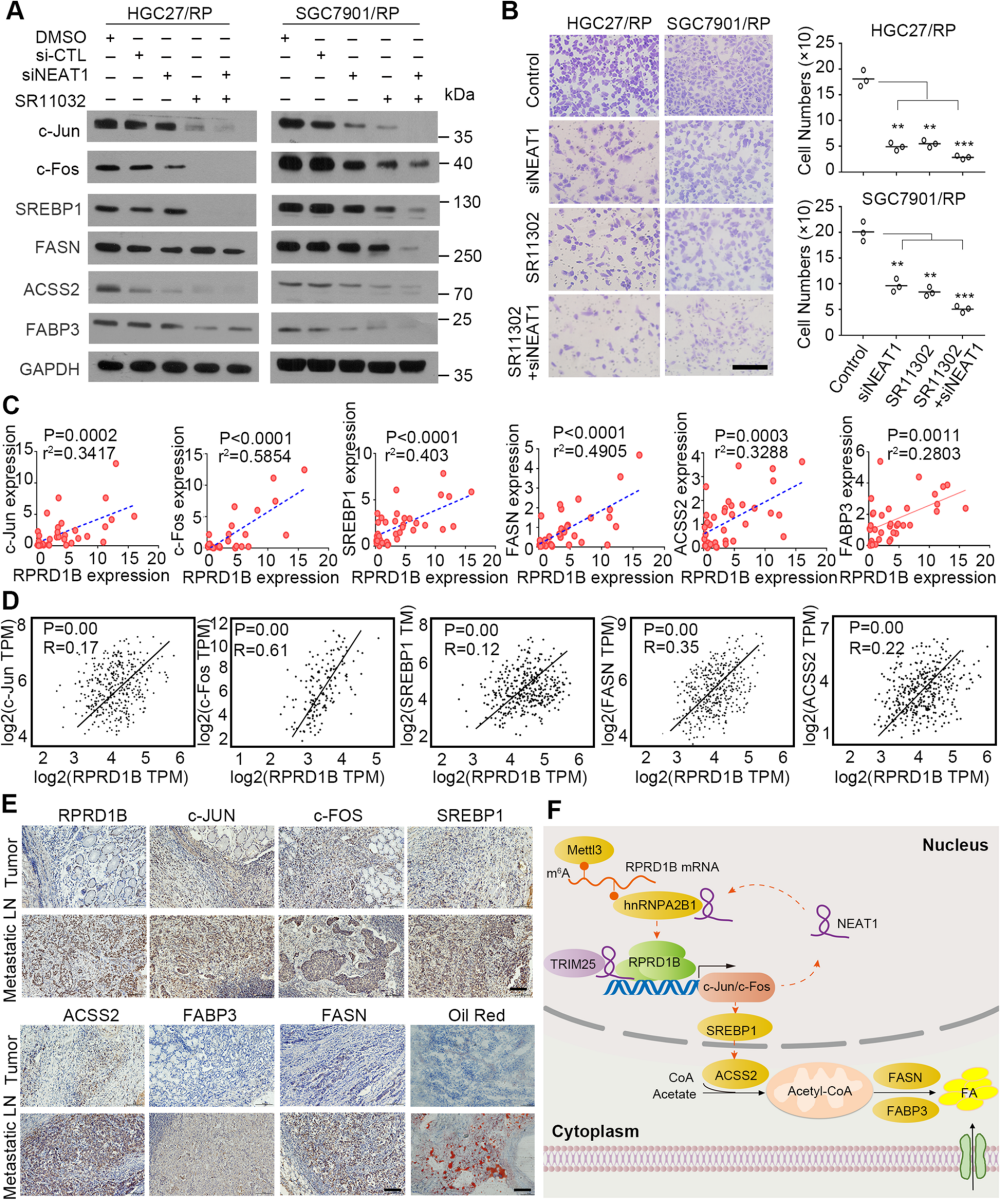
Illustration of the role of the RPRD1B/c-Jun/c-Fos/SREBP1 axis in the lymph node metastasis of GC. **A** The expression of c-Jun, c-Fos, SREBP1, FASN, ACSS2 and FABP3 was reduced following siNEAT1 transfection and SR11302 inhibition. GAPDH was served as loading control. **B** Transwell migration assays showed that SR11302 and siNEAT1 inhibited the RPRD1B-induced migration of HGC27 and SGC7901 cells. Scale bar, 200 μm. **C**, **D** Analyses revealed linear regression curves and significant Pearson’s correlations of RPRD1B expression with c-Jun, c-Fos, SREBP1, FASN, ACSS2 and FABP3 expression in our cohort of GC samples. Positive correlations were confirmed in TCGA data. **E** IHC staining for RPRD1B, c-Jun, c-Fos, SREBP1, FASN, ACSS2 and FABP3 revealed higher levels in metastatic lymph nodes than in primary tumors from patients with GC. Oil Red O staining confirmed the presence of more lipid droplets in metastatic lymph nodes than in primary tumors from patients with GC presenting high RPRD1B expression. Scale bar, 50 μm. **F** Schematic diagram displaying the proposed mechanism of RPRD1B in lymph node metastasis of GC. Data are presented as the means ± SD of three independent experiments. (**, *P* < 0.01, ***, *P* < 0.001)

Cells were treated with siNEAT1 and/or the AP1 inhibitor (SR11302) to further identify the role of the c-Jun/c-Fos/SREBP1 axis and NEAT1-mediated feedback loop in lymph node metastasis promoted by RPRD1B. Western blot analysis showed that siNEAT1 and/or SR11302 significantly decreased the levels of c-Jun, c-Fos, and fatty acid uptake and synthesis markers (SREBP1, FASN, ACSS2 and FABP3) in RPRD1B-overexpressing HGC27 and SGC7901 cells (Fig. 8A). As expected, migration and invasion assays revealed that both SR11302 and siNEAT1 significantly inhibited the migration and invasion of RPRD1B-overexpressing cells compared with control cells (Fig. 8B). Positive correlations between the expression of c-Jun, c-Fos, SREBP1, FASN, ASCC2, FABP3 and RPRD1B at the mRNA level were further observed in 36 pairs of clinical GC samples (Fig. 8C) and then confirmed in the public TCGA database (Fig. 8D). Finally, c-Jun/c-Fos/SREBP1 axis-mediated fatty acid metabolism was verified in RPRD1B-driven metastatic lymph nodes using IHC and Oil Red O staining (Fig. 8E).

## Discussion

The lymph node is considered the first exit for distant organ metastasis and is very important for dissemination, but the underlying mechanism remains elusive. Using RNA sequencing, we compared gene expression profiles among primary tumor tissues, metastatic lesions of lymph nodes and adjacent normal tissues in 4 matched pairs of GC tissues. Combined with the survival analysis of TCGA STAD cohort, we selected 8 target genes associated with lymph node metastasis. As a transcriptional cofactor, RPRD1B was identified as a potential gene initiating lymph node metastasis and selected for further study. The results of q-PCR and IHC confirmed that RPRD1B was one of the most significantly upregulated genes in lymph node metastatic lesions. In the present study, RPRD1B overexpression was detected in 40.8% of primary GCs and 47.3% of metastatic lymph nodes. According to the analysis of clinical features, RPRD1B overexpression was significantly associated with lymph node metastasis, the depth of tumor invasion, vascular invasion and a poorer prognosis. Importantly, higher RPRD1B expression in lymph nodes contributes to shorter survival. Therefore, the underlying mechanism of RPRD1B overexpression in lymph node metastases of patients with GC remains to be elucidated.

Aberrant overexpression of RPRD1B has been reported in many cancers, but the underlying mechanism is unknown. The m^6^A modification has been identified as an important epigenetic and posttranslational regulatory mechanism of pre-mRNA alternative splicing, RNA stability and translation efficiency [34]. Mettl3, an important writer protein, plays key roles in posttranscriptional regulation by affecting RNA stability and translation [35]. Mettl3 sustains the stabilization of the c-MYC mRNA via the m^6^A modification to accelerate tumorigenesis in OSCC [36] and promotes YAP translation to induce drug resistance and metastasis in NSCLC [37]. According to TCGA database, we identified a significant positive correlation between RPRD1B expression and Mettl3. Here, the m^6^A modification induced by Mettl3 also increased the stability of the RPRD1B mRNA.

A series of in vitro and in vivo functional assays revealed that RPRD1B possessed a strong oncogenic function. RPRD1B enhanced tumor cell migration, which is attributable to its effects on lymph node metastasis. We next attempted to explore the molecular mechanism underlying the pro-metastatic effect of RPRD1B. In previous studies, RPRD1B was identified as an oncogene that promotes proliferation by upregulating cell cycle-related genes in various tumors [8]. Here, we describe a novel mechanism by which RPRD1B promotes lymph node metastasis by facilitating fatty acid metabolism via the c-Jun/c-Fos/SREBP1 axis. As a transcriptional coactivator, RPRD1B upregulates the expression of c-Jun and c-Fos at the transcriptional level by directly binding to both promoters. Extensive evidence suggests that the c-Jun/c-Fos/AP1 protein complex coordinates multiple gene expression programs required for metastatic behavior [38, 39].

The AP-1 protein complex is a dimeric leucine zipper (bZIP) transcription factor formed from three different Jun proteins (c-Jun, JunB, and JunD) and four different Fos proteins (c-Fos, FosB, Fra-1, and Fra-2) [40]. Jun and Fos proteins either form homodimers or heterodimers with each other and form heterodimers with other bZIP transcription factors, such as MAF and ATF family members [41]. Therefore, the wide variety of AP-1 dimers have different molecular mechanisms and biological functions, including inflammation, development, and cancer [42]. For example, AP1 proteins, such as Fra-1 and Fra-2, inhibit the PPARγ pathway and reduce the hepatic lipid content. In contrast, other AP-1 proteins, such as c-Fos and JunD, induce hepatic PPARγ signaling and lipid accumulation [43]. Thus, AP1 proteins are critical for the homeostasis of lipid metabolism. Our analysis provided additional evidence that c-Jun and c-Fos proteins form AP1 heterodimers that substantially contribute to lymph node metastasis by inducing fatty acid accumulation in GC cells.

In addition to c-Jun and c-Fos, sterol regulatory element-binding protein 1 (SREBP1) was upregulated in RPRD1B-overexpressing GC cells. SREBP1 is well known as the master regulator of fatty acid and triacylglycerol synthesis and functions similar with PPARγ [44]. Previous studies indicated that AP1 family members such as c-Jun and JNK, activated SREBP1 and increased lipid accumulation in nonalcoholic fatty liver disease (NAFLD) [24, 25]. In the present study, we reported RPRD1B activated the c-Jun/c-Fos/SREBP1 axis and upregulated fatty acid metabolism-associated genes (ACSS2, FASN, and FABP3). Metabolic heterogeneity among primary cancer cells regulates metastatic efficiency and organotropism [45]. A subpopulation of GC cells expressing RPRD1B initiates fatty acid metabolism and induces lymph node metastasis at high efficiency. This hypothesis was confirmed by the results of the AP1 protein inhibitor (SR11302) treatment, as the compound significantly inhibited metastasis- and fatty acid metabolism-related gene expression. Therefore, RPRD1B promotes lymph node metastasis in a c-Jun/c-Fos-dependent manner.

In recent years, extensive dysregulation of lncRNAs in human diseases, including cancer, has attracted increasing attention [46, 47]. Our analysis showed that the lncRNA NEAT1 was dramatically upregulated by the c-Jun/c-Fos transcriptional complex in RPRD1B-overexpressing GC cells. NEAT1 was originally identified in subnuclear organelles called paraspeckles that are free of chromatin and function as repositories of edited RNA and a number of nuclear RNA-binding proteins [48]. Under certain transcriptionally active conditions, NEAT1 is redistributed in the nucleus and recruited to the promoters of several cancer-related target genes to induce an active chromatin state for transcription [29]. Emerging roles for lncRNAs in tumor metabolism through the regulation of metabolic reprogramming have been documented [49]. NEAT1 was reported to regulate ATGL expression by binding to miR-124-3p during the abnormal lipid metabolism of hepatocellular carcinoma [50]. However, the mechanism of NEAT1 in lymph node metastasis and fatty acid metabolism must be illuminated. In the present study, we identified a novel mechanism by which NEAT1 increased the stability of the RPRD1B mRNA by interacting with the nuclear m^6^A reader HNRNPA2B1 and contributed to fatty acid metabolism in RPRD1B-overexpressing GC cells.

In addition, NEAT1 physically interacts with RPRD1B and attenuates RPRD1B degradation mediated by ubiquitin through the retention of RPRD1B in the nucleus. The E3 ubiquitin ligase TRIM25 has been reported to play important roles in the development of several cancers and innate immunity [51]. TRIM25 also regulates adipocyte differentiation via –the proteasomal degradation of PPARγ [52]. As an RNA-binding protein, TRIM25 ubiquitinates proteins in a lncRNA-modulated manner [53]. In the present study, we found that TRIM25 interacted with both NEAT1 and RPRD1B. NEAT1 inhibited the ubiquitination of RPRD1B by preventing its interaction with TRIM25. These results thus indicated that NEAT1 induces RPRD1B expression through a positive feedback loop mediated by the complex RPRD1B-c-Jun/c-Fos/AP1-NEAT1 in GC pathogenesis. This evidence is the first to show that RPRD1B overexpression promotes lymph node metastasis through a lncRNA-mediated positive feedback loop.

## Conclusion

Overall, our study identified m^6^A-induced RPRD1B overexpression as an initiating event in GC lymph node metastasis. RPRD1B further activated the c-Jun/c-Fos/SREBP1 axis, upregulated a cluster of genes related to fatty acid uptake and synthesis (ACSS2, FASN, and FABP3), and facilitated GC cell implantation in lymph nodes (Fig. 8F). These findings provide new insights into the importance of the orchestrated interactions between transcription factors, lncRNAs, and proteins in the lymph node metastasis of GC. A better understanding of the oncogenic mechanisms of RPRD1B in lymph node metastasis may lead to the development of novel therapeutic strategies for GC.

## Supplementary Information

**Additional file 1: Supplementary Table 1.** List of antibodies used in this project. **Supplementary Table 2.** List of PCR primers for expression and cloning. **Supplementary Table 3.** List of PCR primers for Luciferase assay. **Supplementary Table 4.** List of PCR primers for ChIP assay. **Supplementary Table 5.** List of probes for EMSA assay. **Supplementary Table 6.** Association of RPRD1B upregulation with clinicopathologic features in 191 GCs. **Supplementary Fig. 1.** (A) RT–qPCR showing that RPRD1B was the most significantly overexpressed gene in 8 target genes in both GC tissue and metastatic lymph node (*n* = 10). (B, C) Levels of the RPRD1B and Mettl3 proteins and mRNAs after Mettl3 knockdown or overexpression in HGC27 cells. (D) RIP-qPCR showing the enrichment of m^6^A in HGC27 cells after Mettl3 depletion, independent Student’s t test. (E) The decay rate of the RPRD1B mRNA after treatment with 2.5 μM actinomycin D for the indicated times in AGS cells with Mettl3 knockdown or overexpression. GAPDH was served as the loading control. Data are presented as the means ± SD of three independent experiments. (*, *P* < 0.05; ***, *P* < 0.001). **Supplementary Fig. 2.** (A, B) Wound-healing assay showing that RPRD1B overexpression promoted the migration of SGC7901 cells and RPRD1B knockdown inhibited the migration of BGC823 cells at 0, 24, and 48 h after scratch wounding. (C) Transwell migration assay showing that SR11302 inhibited the RPRD1B-induced migration of SGC7901 cells. Scale bar, 200 μm. (D) Transwell migration assay showed that NEAT1 was upregulated in RPRD1B-overexpressing HGC27 and SGC7901 cells. The effect was diminished by SR11302. (E) NEAT1 was downregulated in AGS and BGC823 RPRD1B-silenced cells and rescued by c-Jun and c-Fos. Scale bar, 20 μm. Data are presented as the means ± SD of three independent experiments. (NS, not significant; **, *P* < 0.01; ***, *P* < 0.001). **Supplementary Fig. 3.** (A) CoIP assay validated that hnRNPA2B1, not YTHDF1, directly interacted with NEAT1 in HGC27 cells. (B) Levels of the hnRNPA2B1 and RPRD1B proteins and mRNAs after RPRD1B inhibition in RPRD1B-overexpressing SGC7901 cells. The results are summarized as the means ± SD of three independent experiments. (C) MeRIP-qPCR showing the enrichment of m^6^A in HGC27 cells after hnRNPA2B1 depletion. (D) The decay rate of the RPRD1B mRNA after treatment with 2.5 μM actinomycin D for the indicated times following hnRNPA2B1 knockdown in RPRD1B-overexpressing HGC27 cells. (E) RIP-qPCR showing the enrichment of hnRNPA2B1 on the RPRD1B mRNA in RPRD1B-overexpressing HGC27 cells with NEAT1 silencing. (F) The IHC staining and Oil red O staining were performed in 10 cases of GC cohort with RPRD1B overexpression. The IHC score were summarized. GAPDH was served as the loading control. Data are presented as the means ± SD of three independent experiments. (**, *P* < 0.01; ***, *P* < 0.001). **Supplementary Fig. 4.** Preliminary IHC staining showed the optimum concentration of antibody and verified the correctness and specificity of antibody.

## Data Availability

All data generated or analyzed during this study are included in this published article and supplementary information files.
