# Supplementary material for: Long non-coding RNA NEAT1 mediated RPRD1B stability facilitates fatty acid metabolism and lymph node metastasis via c-Jun/c-Fos/SREBP1 axis in gastric cancer
Source: J Exp Clin Cancer Res. 2022 Sep 29;41:287. doi: 10.1186/s13046-022-02449-4 (PMC9520879; doi:10.1186/s13046-022-02449-4)
Supplement: Supplementary file 1 — Additional file 1: Supplementary Table 1. List of antibodies used in this project. Supplementary Table 2. List of PCR primers for expression and cloning. Supplementary Table 3. List of PCR primers for Luciferase assay. Supplementary Table 4. List of PCR primers for ChIP assay. Supplementary Table 5. List of probes for EMSA assay. Supplementary Table 6. Association of RPRD1B upregulation with clinicopathologic features in 191 GCs. Supplementary Fig. 1. (A) RT–qPCR showing that RPRD1B was the most significantly overexpressed gene in 8 target genes in both GC tissue and metastatic lymph node (n = 10). (B, C) Levels of the RPRD1B and Mettl3 proteins and mRNAs after Mettl3 knockdown or overexpression in HGC27 cells. (D) RIP-qPCR showing the enrichment of m6A in HGC27 cells after Mettl3 depletion, independent Student’s t test. (E) The decay rate of the RPRD1B mRNA after treatment with 2.5 μM actinomycin D for the indicated times in AGS cells with Mettl3 knockdown or overexpression. GAPDH was served as the loading control. Data are presented as the means ± SD of three independent experiments. (*, P < 0.05; ***, P < 0.001). Supplementary Fig. 2. (A, B) Wound-healing assay showing that RPRD1B overexpression promoted the migration of SGC7901 cells and RPRD1B knockdown inhibited the migration of BGC823 cells at 0, 24, and 48 h after scratch wounding. (C) Transwell migration assay showing that SR11302 inhibited the RPRD1B-induced migration of SGC7901 cells. Scale bar, 200 μm. (D) Transwell migration assay showed that NEAT1 was upregulated in RPRD1B-overexpressing HGC27 and SGC7901 cells. The effect was diminished by SR11302. (E) NEAT1 was downregulated in AGS and BGC823 RPRD1B-silenced cells and rescued by c-Jun and c-Fos. Scale bar, 20 μm. Data are presented as the means ± SD of three independent experiments. (NS, not significant; **, P < 0.01; ***, P < 0.001). Supplementary Fig. 3. (A) CoIP assay validated that hnRNPA2B1, not YTHDF1, directly interacted with NEAT1 in HGC27 ce [file 13046_2022_2449_MOESM1_ESM.docx]

**Supplementary Information**

**Supplementary Table 1. List of antibodies used in this project**

| Antibody | Cat No. | Vendor | Application |
| --- | --- | --- | --- |
| Rabbit anti-human RPRD1B | NBP2-20222 | Novus | WB, 1:1000; IHC, 1:1000 |
| Rabbit anti-human RPRD1B | Ab137246 | Abcam | IP/CHIP: 1:50 |
| Rabbit anti-human c-Jun | 9165 | CST | WB, 1:1000; CHIP, 1:50; IHC, 1:200 |
| Rabbit anti-human c-Fos | 2250 | CST | WB, 1:1000; CHIP, 1:50; IHC, 1:200 |
| Rabbit anti-human SREBP1 | 140881-AP | Proteintech | WB, 1:1000; IHC, 1:200 |
| Rabbit anti-human FASN | 3180 | CST | WB, 1:1000; IHC, 1:200 |
| Rabbit anti-human ACSS2 | GTX30020 | Genetex | WB, 1:1000; IHC, 1:200 |
| Rabbit anti-human FABP3 | 60280-1-Ig | Proteintech | WB, 1:1000; IHC, 1:200 |
| Rabbit anti-human TRIM25 | NBP1-00113 | Novus | WB, 1:1000; IP, 1:50 |
| Rabbit anti-human Ubiquitin | 3933 | CST | WB, 1:1000 |
| Rabbit anti-human m6A | A-1801-100 | EpiGentek | IP: 1:50 |
| Rabbit anti-human β-actin | 4970 | CST | WB, 1:1000 |
| Rabbit anti-human GAPDH | 5174 | CST | WB, 1:1000 |

**Supplementary Table 2. List of PCR primers for expression and cloning**

| Gene Name | Primer Sequence (5’-3’) |
| --- | --- |
| **For Real-time PCR** |  |
| *RPRD1B* | Forward: CACCCGGTAAAAAGTCCCG |
| NM_001323982.1 | Reverse: AGGCTCCAACAGTCTCTCAAC |
| *FASN* | Forward: AAGGACCTGTCTAGGTTTGATGC |
| NM_004104 | Reverse: TGGCTTCATAGGTGACTTCCA |
| *SREBP1* | Forward: CGGAACCATCTTGGCAACAGT |
| NM_001005291 | Reverse: CGCTTCTCAATGGCGTTGT |
| *ACSS2* | Forward: AAAGGAGCAACTACCAACATCTG |
| NM_001242393 | Reverse: GCTGAACTGACACACTTGGAC |
| *FABP3* | Forward: TGGAGTTCGATGAGACAACAGC |
| NM_004102 | Reverse: CTCTTGCCCGTCCCATTTCTG |
| *c-Jun* | Forward: TGAGTGACCGCGACTTTTCA |
| NM_002228.4 | Reverse: TTTCTCTAAGAGCGCACGCA |
| *c-Fos* | Forward: AACCGCCACGATGATGTTCT |
| NM_005252.4 | Reverse: TCTGCGGGTGAGTGGTAGTA |
| *NEAT1* | Forward: CTGGTCATCTGGTAAGCCCG |
| NR_028272.1 | Reverse: ACATTCACTCCCCACCCTCT |
| β-actin | Forward: CGGCGCCCTATAAAACCCA |
| NM_001101.4 | Reverse: CGCGGCGATATCATCATCCA |
| *GAPDH* | Forward: GGAGCGAGATCCCTCCAAAAT |
| NM_001256799 | Reverse: GGCTGTTGTCATACTTCTCATGG |
| **For full length cDNA** |  |
| RPRD1B-v5 | Forward: caccCTGTCAGTCGGTAAAAAGTCC |
|  | Reverse: GTTGAAAACAGGTCCCCAGCAGAG |
| NEAT1 | Forward: caccAGTTAGCGACAGGGAGGGAT |
|  | Reverse: AGTTTGAGTTCTAAACTCATTAG |

**Supplementary Table 3. List of PCR primers for Luciferase assay**

| Gene Name | Primer Sequence (5’-3’) |
| --- | --- |
| **For Luciferase assay** |  |
| c-Jun-Fragment 1 | Forward: taaggtaccGCAACAGACAGAGGAGAATGT |
|  | Reverse: taaaagcttGAGCTCAACACTTATCTGCTA |
| c-Jun-Fragment 2 | Forward: taaggtaccTATCCAGGCTCTGCGAGGATG |
|  | Reverse: taaaagcttGAGCTCAACACTTATCTGCTA |
| c-Jun-Fragment 3 | Forward: taaggtaccGTGTTAAGCGTGTGCGTGTTG |
|  | Reverse: taaaagcttGAGCTCAACACTTATCTGCTA |
| c-Jun-Fragment 4 | Forward: taaggtaccGACTTCACAGAGCCACCTTAA |
|  | Reverse: taaaagcttGAGCTCAACACTTATCTGCTA |
| c-Fos-Fragment 1 | Forward: taaggtaccACCAATGACATACAATGATGA |
|  | Reverse: taaaagcttCCTCGTAGTCTGCGTTGAAGC |
| c-Fos-Fragment 2 | Forward: taaggtaccCTGGAGGCAGGTCTGCGGTCC |
|  | Reverse: taaaagcttCCTCGTAGTCTGCGTTGAAGC |
| c-Fos-Fragment 3 | Forward: taaggtaccCTCCCGTAAGCACAGCTTCCT |
|  | Reverse: taaaagcttCCTCGTAGTCTGCGTTGAAGC |
| c-Fos-Fragment 4 | Forward: taaggtaccTATCTCTGAGCCTCAGAACTG |
|  | Reverse: taaaagcttCCTCGTAGTCTGCGTTGAAGC |
| NEAT1 | Forward: taaggtaccAGGCTGGTCTCGAACTCCTGG |
|  | Reverse: taaaagcttTAGCCCTCAGCCGCGTCACCG |

**Supplementary Table 4. List of PCR primers for ChIP assay**

| Gene Name | Primer Sequence (5’-3’) |
| --- | --- |
| **For ChIP assay** |  |
| c-Jun-Primer 1 | Forward: GCAACAGACAGAGGAGAATGT |
|  | Reverse: TGAGTGTTCAGAAACCAACAG |
| c-Jun-Primer 2 | Forward: TATCCAGGCTCTGCGAGGATG |
|  | Reverse: TTCGTGGCCAGGCTACCACAG |
| c-Jun-Primer 3 | Forward: GTGTTAAGCGTGTGCGTGTTG |
|  | Reverse: TGGACGCTCAGCTGAGTCTCC |
| c-Jun-Primer 4 | Forward: GACTTCACAGAGCCACCTTAA |
|  | Reverse: GCCAGGGAACCTGGCCAGTTG |
| c-Fos-Primer 1 | Forward: ATAGCAAGTGGCCCAGTTCCA |
|  | Reverse: GCCACCCTCCCTCCCAGCTCC |
| c-Fos-Primer 2 | Forward: CTGGAGGCAGGTCTGCGGTCC |
|  | Reverse: AGCGCGCTGGCCACCTCCAGG |
| c-Fos-Primer 3 | Forward: CTCCCGTAAGCACAGCTTCCT |
|  | Reverse: ACCAGCAGATAAACACTGTGC |
| c-Fos-Primer 4 | Forward: TATCTCTGAGCCTCAGAACTG |
|  | Reverse: GGCTGCAGCCAACACCGAGGG |

**Supplementary Table 5. List of probes for EMSA assay**

| Gene Name | Probe Sequence (5’-3’) | Label |
| --- | --- | --- |
| **For EMSA assay** |  |  |
| c-Jun-Fragment 1 | Forward: GCAACAGACAGAGGAGAATGT | 3’ Biotin |
|  | Reverse: TGAGTGTTCAGAAACCAACAG | 3’ Biotin |
| c-Jun-Fragment 2 | Forward: TATCCAGGCTCTGCGAGGATG | 3’ Biotin |
|  | Reverse: TTCGTGGCCAGGCTACCACAG | 3’ Biotin |
| c-Jun- Fragment 3 | Forward: GTGTTAAGCGTGTGCGTGTTG | 3’ Biotin |
|  | Reverse: TGGACGCTCAGCTGAGTCTCC | 3’ Biotin |
| c-Jun- Fragment 4 | Forward: GACTTCACAGAGCCACCTTAA | 3’ Biotin |
|  | Reverse: GCCAGGGAACCTGGCCAGTTG | 3’ Biotin |
| c-Fos- Fragment 1 | Forward: ATAGCAAGTGGCCCAGTTCCA | 3’ Biotin |
|  | Reverse: GCCACCCTCCCTCCCAGCTCC | 3’ Biotin |
| c-Fos- Fragment 2 | Forward: CTGGAGGCAGGTCTGCGGTCC | 3’ Biotin |
|  | Reverse: AGCGCGCTGGCCACCTCCAGG | 3’ Biotin |
| c-Fos- Fragment 3 | Forward: CTCCCGTAAGCACAGCTTCCT | 3’ Biotin |
|  | Reverse: ACCAGCAGATAAACACTGTGC | 3’ Biotin |
| c-Fos- Fragment 4 | Forward: TATCTCTGAGCCTCAGAACTG | 3’ Biotin |
|  | Reverse: GGCTGCAGCCAACACCGAGGG | 3’ Biotin |

**Supplementary Table 6. Association of RPRD1B upregulation with clinicopathologic features in 191 GCs**

| Features | Total | RPRD1B expression | | P value |
| --- | --- | --- | --- | --- |
|  |  | Low expression | High expression |  |
| Gender |  |  |  |  |
| Male | 153 | 88 | 65 |  |
| Female | 38 | 25 | 13 | 0.461 |
| Age, y |  |  |  |  |
| <60 | 106 | 59 | 47 |  |
| >60 | 85 | 54 | 31 | 0.302 |
| Tumor location |  |  |  |  |
| Cadia | 32 | 16 | 16 |  |
| Body | 140 | 86 | 54 |  |
| Antrum | 19 | 11 | 8 | 0.491 |
| Histopathology |  |  |  |  |
| Tubular adenocarcinoma | 157 | 85 | 72 |  |
| Mucous adenocarcinoma | 22 | 18 | 4 |  |
| Signet ring carcinoma | 11 | 9 | 2 |  |
| Squamous carcinoma | 1 | 1 | 0 | 0.276 |
| Differentiation |  |  |  |  |
| Well | 94 | 61 | 33 |  |
| Moderate | 50 | 24 | 26 |  |
| Poor | 47 | 28 | 19 | 0.145 |
| Depth of tumor |  |  |  |  |
| T1,2 | 65 | 46 | 19 |  |
| T3,4 | 126 | 67 | 59 | **0.019** |
| Lymphnode invasion |  |  |  |  |
| Absent | 43 | 33 | 10 |  |
| Present | 81 | 44 | 37 | **0.014** |
| Vascular invasion |  |  |  |  |
| Absent | 50 | 38 | 12 |  |
| Present | 28 | 10 | 18 | **0.000** |

**Supplementary Figures**


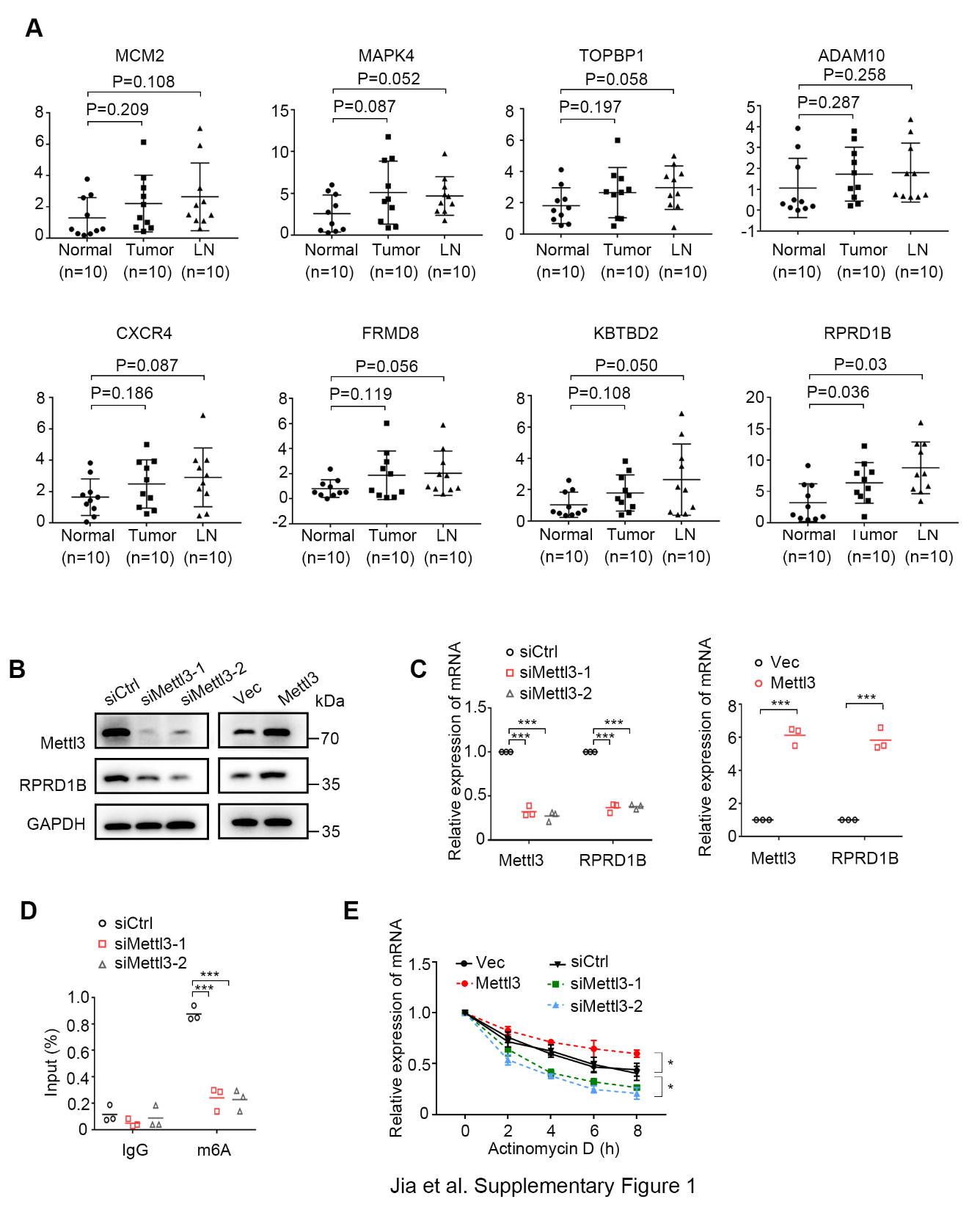


**Supplementary Figure 1.** (A) RT–qPCR showing that RPRD1B was the most significantly overexpressed gene in 8 target genes in both GC tissue and metastatic lymph node (n = 10). (B, C) Levels of the RPRD1B and Mettl3 proteins and mRNAs after Mettl3 knockdown or overexpression in HGC27 cells. (D) RIP-qPCR showing the enrichment of m^6^A in HGC27 cells after Mettl3 depletion, independent Student’s t test. (E) The decay rate of the RPRD1B mRNA after treatment with 2.5 μM actinomycin D for the indicated times in AGS cells with Mettl3 knockdown or overexpression. GAPDH was served as the loading control. Data are presented as the means ± SD of three independent experiments. (*, P < 0.05; ***, P < 0.001).


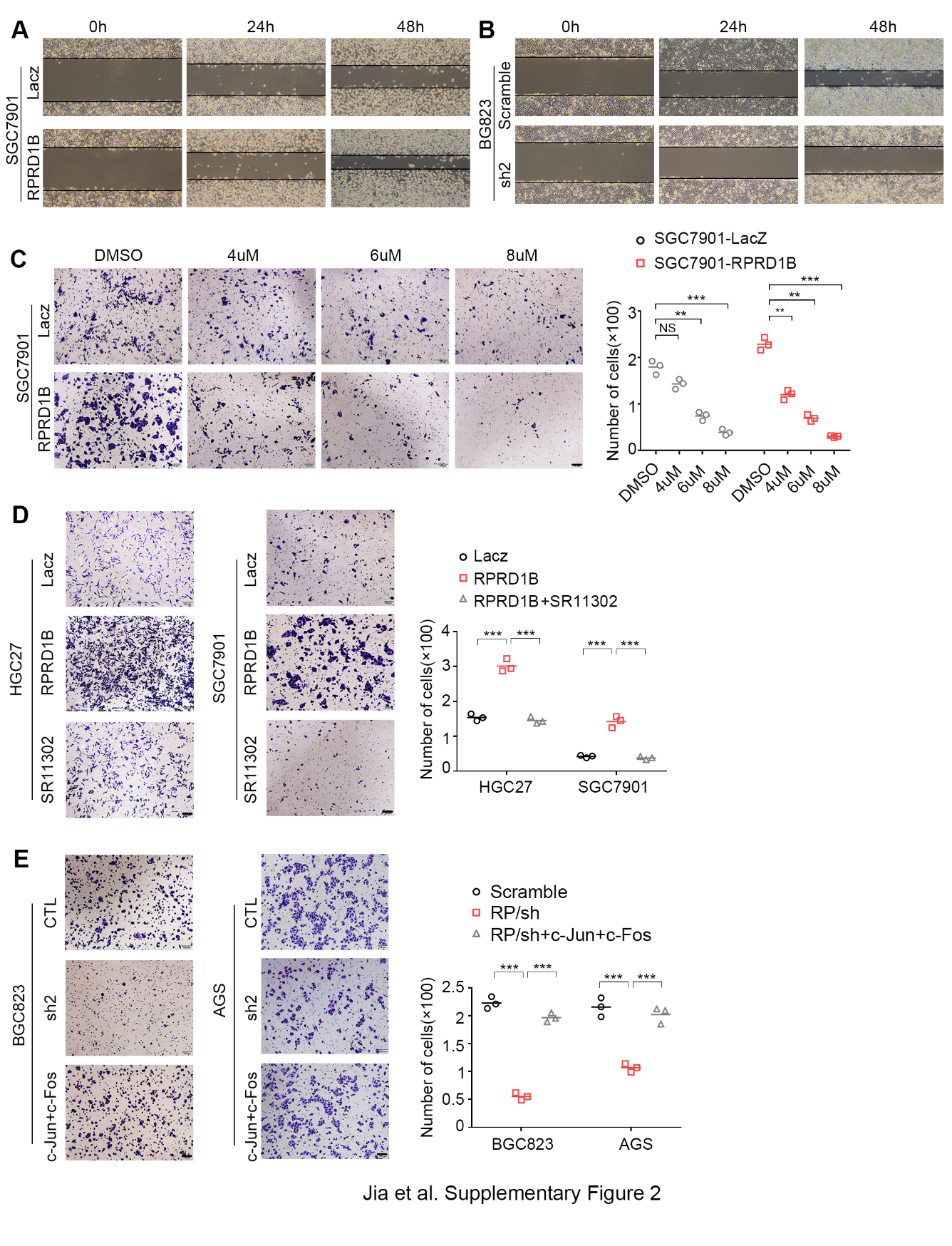


**Supplementary Figure 2.** (A, B) Wound-healing assay showing that RPRD1B overexpression promoted the migration of SGC7901 cells and RPRD1B knockdown inhibited the migration of BGC823 cells at 0, 24, and 48 h after scratch wounding. (C) Transwell migration assay showing that SR11302 inhibited the RPRD1B-induced migration of SGC7901 cells. Scale bar, 200 μm. (D) Transwell migration assay showed that NEAT1 was upregulated in RPRD1B-overexpressing HGC27 and SGC7901 cells. The effect was diminished by SR11302. (E) NEAT1 was downregulated in AGS and BGC823 RPRD1B-silenced cells and rescued by c-Jun and c-Fos. Scale bar, 20 μm. Data are presented as the means ± SD of three independent experiments. (NS, not significant; **, P < 0.01; ***, P < 0.001).


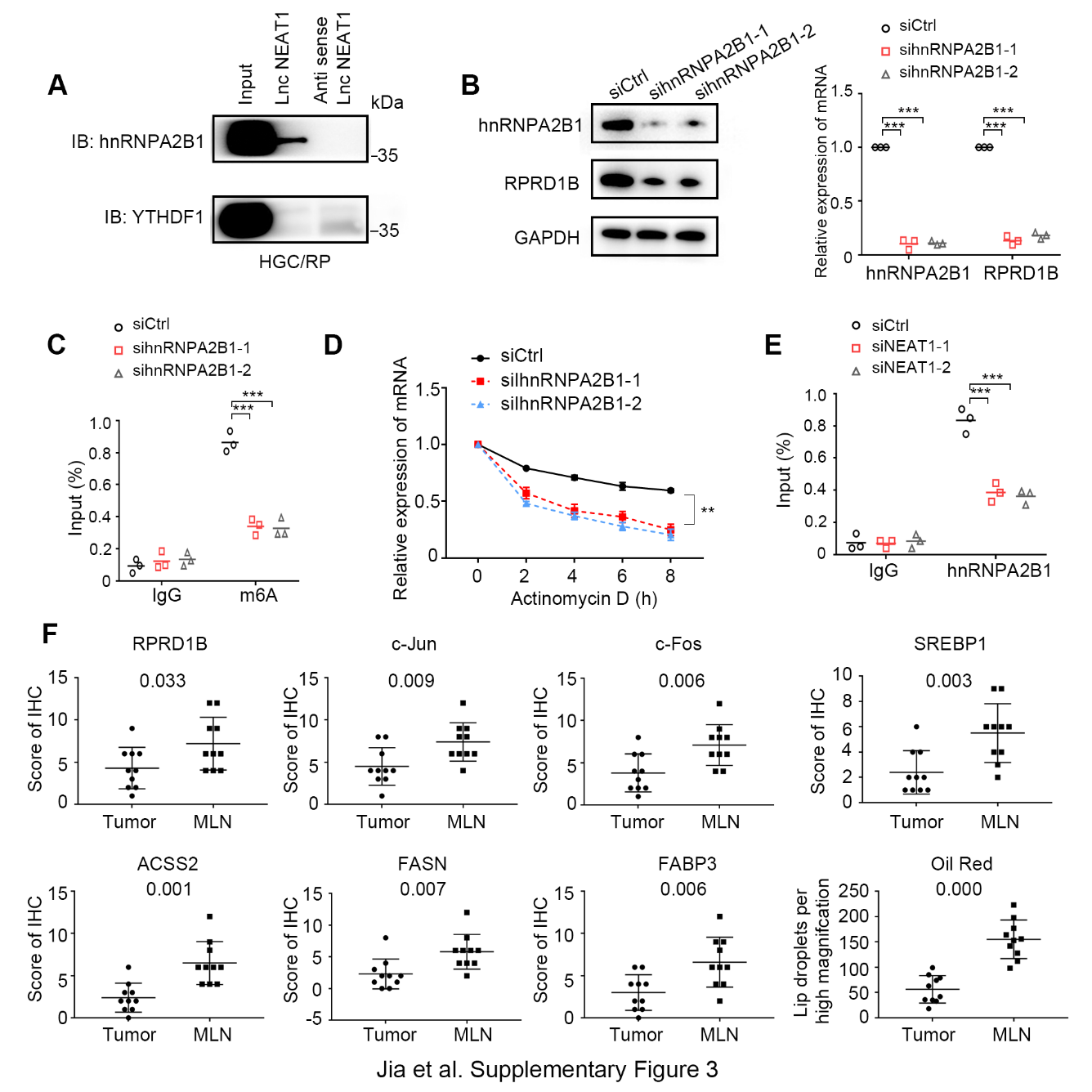


**Supplementary Figure 3.** (A) CoIP assay validated that hnRNPA2B1, not YTHDF1, directly interacted with NEAT1 in HGC27 cells. (B) Levels of the hnRNPA2B1 and RPRD1B proteins and mRNAs after RPRD1B inhibition in RPRD1B-overexpressing SGC7901 cells. The results are summarized as the means ± SD of three independent experiments. (C) MeRIP-qPCR showing the enrichment of m^6^A in HGC27 cells after hnRNPA2B1 depletion. (D) The decay rate of the RPRD1B mRNA after treatment with 2.5 μM actinomycin D for the indicated times following hnRNPA2B1 knockdown in RPRD1B-overexpressing HGC27 cells. (E) RIP-qPCR showing the enrichment of hnRNPA2B1 on the RPRD1B mRNA in RPRD1B-overexpressing HGC27 cells with NEAT1 silencing. (F) The IHC staining and Oil red O staining were performed in 10 cases of GC cohort with RPRD1B overexpression. The IHC score were summarized. GAPDH was served as the loading control. Data are presented as the means ± SD of three independent experiments. (**, P < 0.01; ***, P < 0.001).


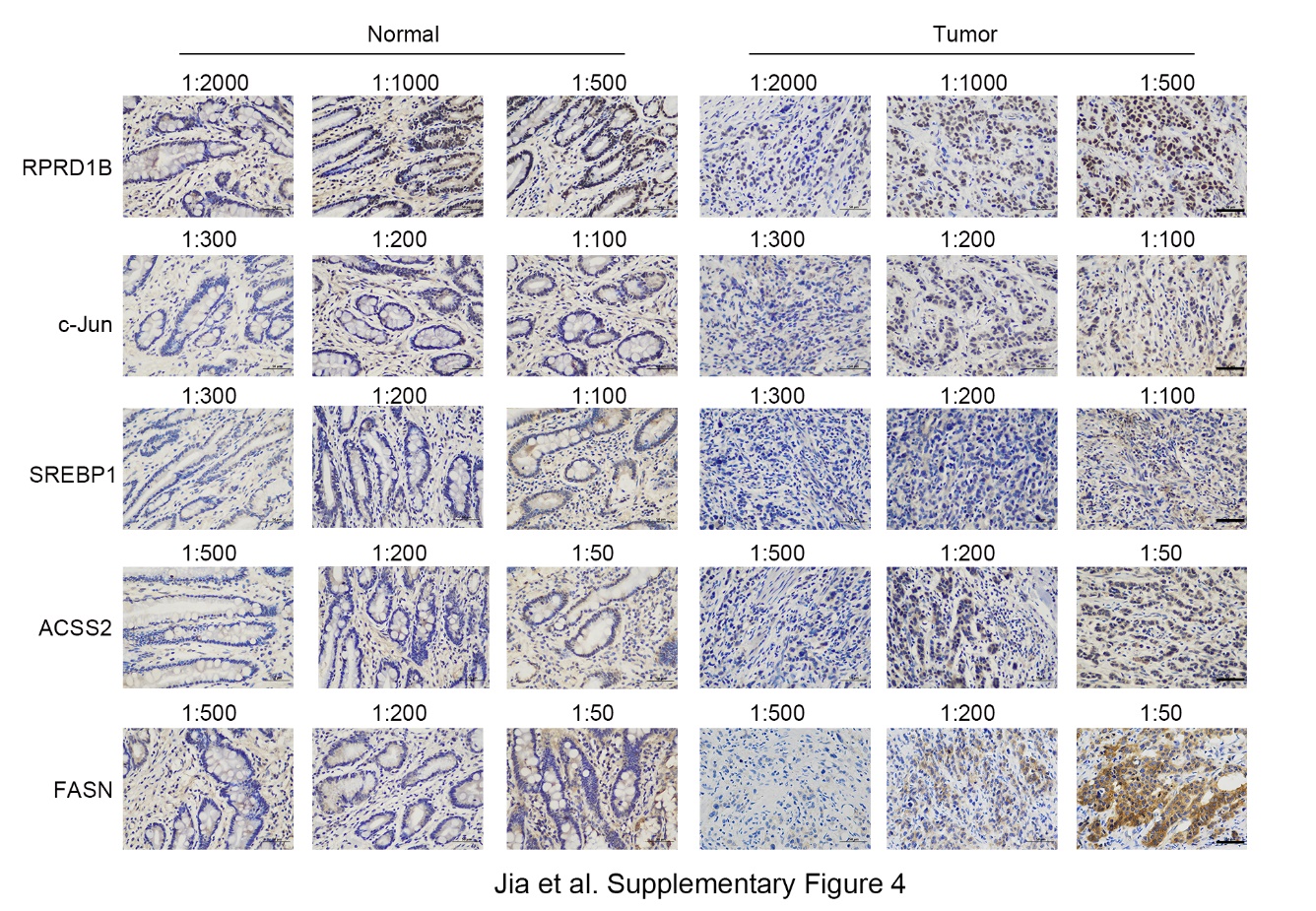


**Supplementary Figure 4.** Preliminary IHC staining showed the optimum concentration of antibody and verified the correctness and specificity of antibody.
